# Impact of iatrogenic alterations on adjacent segment degeneration after lumbar fusion surgery: a systematic review

**DOI:** 10.1186/s13018-025-05561-1

**Published:** 2025-04-29

**Authors:** Moritz Jokeit, Christos Tsagkaris, Franziska C. S. Altorfer, Frédéric Cornaz, Jess G. Snedeker, Mazda Farshad, Jonas Widmer

**Affiliations:** 1 Spine Biomechanics, Department of Orthopedics, Balgrist University Hospital, University of Zurich, Lengghalde 5, CH-8008 Zurich, Switzerland; 2 Department of Orthopedics, Balgrist University Hospital, University of Zurich, Zurich, Switzerland; 3 Institute for Biomechanics, ETH Zurich, Zurich, Switzerland

## Abstract

**Purpose:**

Adjacent segment degeneration (ASDeg) and disease (ASDis) remain significant challenges following lumbar spinal fusion surgery, with reported incidences of 36% for ASDeg and 11% for ASDis within two to seven years post-operation. However, the mechanisms leading to the development of ASDeg are still poorly understood. This comprehensive review aims to elucidate the multifactorial etiology of ASDeg by examining important iatrogenic alterations associated with spinal fusion.

**Methods:**

A systematic review following PRISMA guidelines was conducted to identify clinical studies quantifying the occurrence of ASDeg and ASDis after lumbar fusion surgery. An EMBASE and citation search up to April 2023 yielded 378 articles. Data extracted encompassed study design, fusion type, sample size, patient age, and incidence of ASDeg and ASDis. A total of 87 publications were analyzed in the context of iatrogenic alterations caused by surgical access (muscle damage, ligament damage, facet joint damage) and instrumentation (fusion angle, immobilization).

**Results:**

Ligament damage emerged as the most impactful iatrogenic factor promoting ASDeg and ASDis development. Similarly, muscle damage had a significant impact on long-term musculoskeletal health, with muscle-sparing approaches potentially reducing ASDis rates. Immobilization led to compensatory increased motion at adjacent segments; however, the causal link to degeneration remains inconclusive. Fusion angle showed low evidence for a strong impact due to inconsistent findings across studies. Facet joint violations were likely contributing factors but not primary initiators of ASDeg.

**Conclusion:**

Based on the analyzed literature, ligament and muscle damage are the most impactful iatrogenic factors contributing to ASDeg and ASDis development. Minimally invasive techniques, careful retractor placement, and ligament-preserving decompression may help mitigate these effects by reducing undue muscle and ligament trauma. Although it is not possible to definitively advocate for one or more techniques, the principle of selecting the most tissue-sparing approach needs to be scaled across surgical planning and execution. Further research is necessary to fully elucidate these mechanisms and inform surgical practices to mitigate ASDeg risk.

## Introduction

When conservative treatment options fail, spinal fusion remains the standard surgical procedure for the majority of back pain-related disorders, especially in the thoracolumbar spine [1, 2]. Although fusion surgery has been associated with substantial improvement in patients’ mobility, pain, overall functionality, and perceived well-being [3], a non-negligible proportion of patients experience complications that require re-operation. The principal complications leading to revision surgery are pseudoarthrosis [4] and adjacent segment degeneration. Adjacent segment degeneration (ASDeg) is an umbrella term to describe postoperative *radiographic changes*, e.g., herniated discs, facet joint hypertrophy, and endplate defects in the adjacent segment, and the new onset of *clinical symptoms,* termed adjacent segment disease (ASDis) [5]. According to a recent meta-analysis of 19 studies including 719 fusion patients with a follow-up of 29 to 92 months, spinal fusion was associated with an incidence of 36% for ASDeg, 11% for ASDis, and 7% for ASDis related revision surgery [6]. Another meta-analysis of 31 studies including 4206 patients, reported a pooled incidence of 5.9% per year for ASDeg and 1.8% per year for ASDis [7].

These findings indicate that approximately one-third of patients develop at least radiographic ASDeg, one-tenth of patients experience symptoms in form of ASDis, and more than one-twentieth of patients undergo reoperation within five years or more. The ever-increasing rate of fusion surgeries [8, 9] leads to proportionately increased rates and burden of ASDeg. Therefore, understanding the causes of ASDeg and ASDis and translating them into prevention and damage-control strategies in the operating theater and beyond is of paramount importance. Despite being the subject of extensive research [10, 11] with well over 5000 publications on the topic, the multifactorial etiology of ASDeg and ASDis is still not fully understood and remains controversial [5, 12, 13].

This is because the fusion intervention and its effect on the spinal system is highly complex. The clinical indications for fusion surgery include low back pain related to intervertebral disc (IVD) or facet joint disruption, lumbar spine degeneration in the context of degenerative scoliosis and symptomatic spondylolisthesis, radiculopathy associated with foraminal stenosis and neurogenic claudication [14]. Spinal fusion immobilizes the affected segment (index level) by promoting a bony connection between two vertebral bodies. The aim is to eliminate painful motion and neural compression and to prevent further segmental degeneration and neurological complications. Implants such as pedicle screws and intervertebral cages are often used to facilitate bone healing.

Various hypotheses have been proposed to explain the development of ASDeg, most of which focus on mechanical alterations [13, 15, 16]. These changes include but are not limited to, immobilization, foreign body insertion, altered loading conditions, or soft tissue impairment. What is lacking is a detailed description of how each iatrogenic alteration affects adjacent level mechanics, the consequences for spinal tissue loading, and their potential to accelerate ASDeg.

**Fig. 1:**
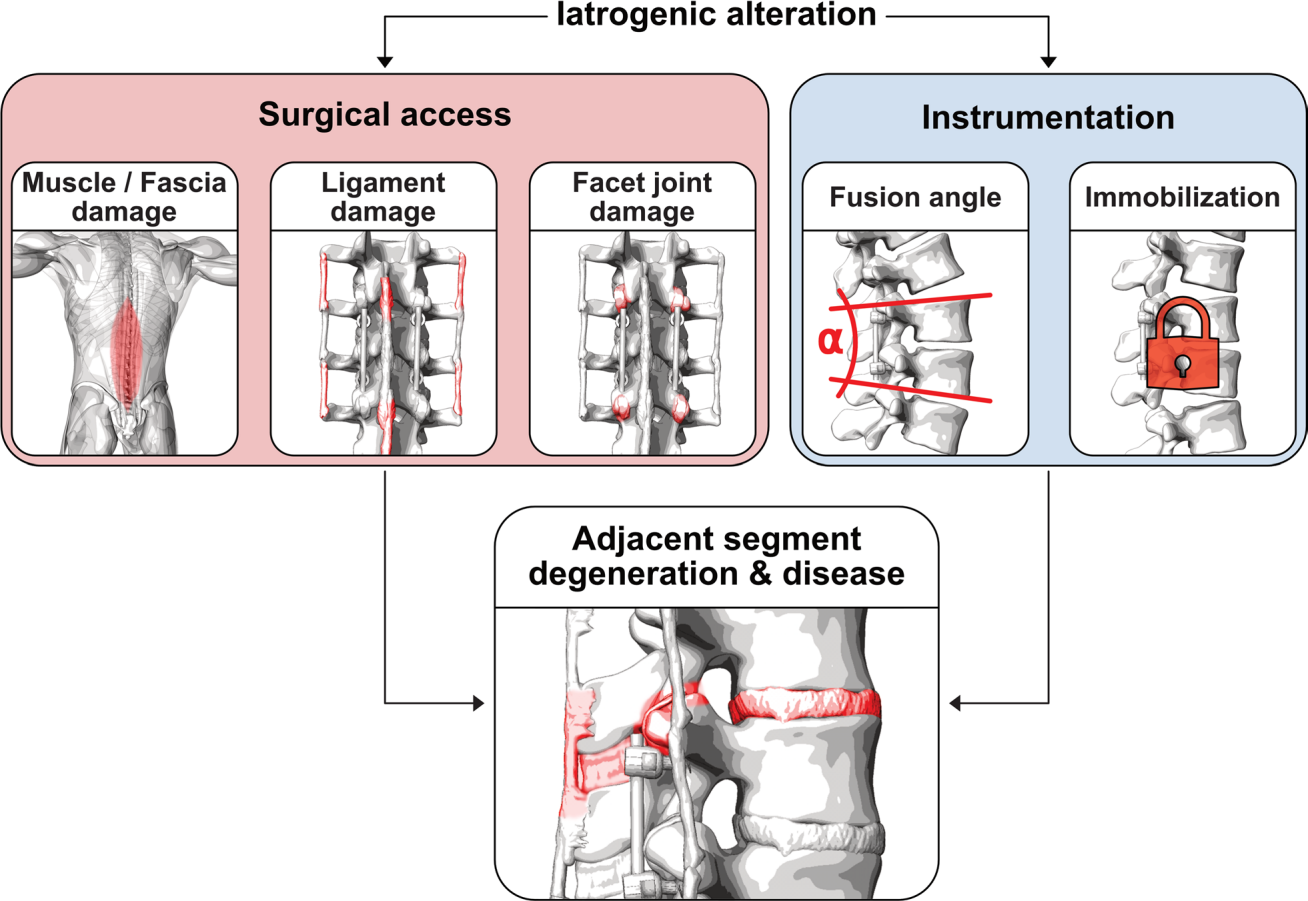
Iatrogenic alterations caused by fusion surgery

The review attempts to identify (and quantify) the effect of the various iatrogenic changes based on the existing literature, and thus, clarify to which extent these factors promote the development of ASDeg. As illustrated in Fig. 1, this review distinguishes between iatrogenic alterations caused by surgical access:

- Muscle (and fascia) damage,
- Ligament damage,
- Facet joint damage,

and iatrogenic change due to instrumentation:

- Fusion angle (impact on alignment),
- Immobilization (kinematic changes).

In this way, clinical and biomechanical researchers are supported in identifying existing research gaps and finding directions for future investigations. Regarding clinical practice, the review aims to open the discussion about how iatrogenic damage, such as muscle or ligament damage, facet joint violation, and immobilization contribute to the development of ASDeg.

## Methods

### Search strategy

**Fig. 2:**
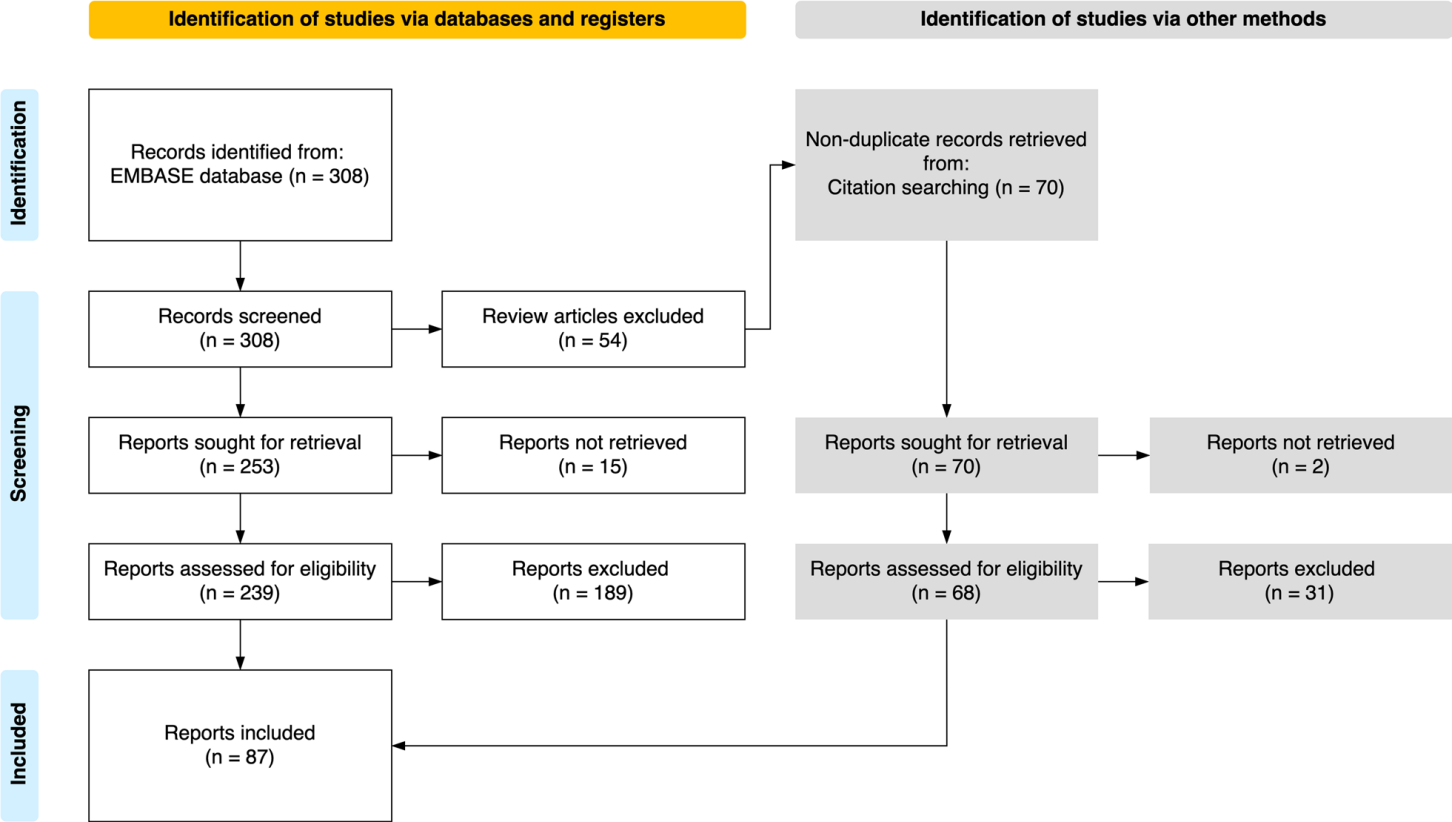
PRISMA flowchart

The review was conducted following the PRISMA guidelines for systematic reviews (Fig. 2) [17]. To identify clinical studies that quantified the occurrence of ASDeg and/or ASDis the authors searched the EMBASE database with a publication cutoff date of April 2023. The EMBASE candidate terms “adjacent segment degeneration”, “adjacent segment disease”, and “adjacent segment pathology” were combined with the terms “incidence”, “prevalence”, and “lumbar” using Boolean operators (AND, OR) where appropriate. The results were filtered to include only English or German publication language and full-text research articles.

### Inclusion and exclusion criteria

The given search criteria resulted in 254 original research reports and 54 review articles which were retrieved for a detailed eligibility check. The reference lists of identified reviews and meta-analyses were searched to determine additional 70 relevant studies. No sample size restriction was applied when screening for eligible studies. Studies were included if they fulfilled the following eligibility criteria:

1. Ongoing or published clinical study reporting on the incidence or the risk of ASDeg or ASDis in the lumbar spine after spinal fusion.
2. One of the following topic-specific outcome measures:
  Muscle damage: comparison of open and minimal-invasive approaches or ventral/lateral and dorsal approaches,
  Ligament damage: comparison of index segment decompression and no/partial index segment decompression or comparison of adjacent segment decompression and no adjacent segment decompression,
  Facet joint violation: comparison of patients with and without facet joint violations,
  Fusion angle: reporting of postoperative segmental lordosis or change in segmental lordosis,
  Immobilization (compensation): reporting of change between pre- and postoperative angular ROM in lumbar spine, upper adjacent or lower adjacent segment,
  Immobilization (fusion length): comparison of short and long fusion constructs.

A study was excluded if it met at least one of the following criteria:

1. Focus on the cervical or thoracic spine,
2. Study types: opinion articles and perspectives,
3. Only motion-preserving interventions,
4. Updated study on same cohort exists,
5. No outcome measure of interest reported.

After the final screening by the first three authors, 87 publications were included in the review and analyzed in the context of one or multiple iatrogenic alterations. Disagreements in the selection of relevant studies were discussed between the first three listed authors and a senior author until a consensus was reached. In addition, topic-specific mini-reviews identified studies on the quantitative extend of muscle damage and facet joint violations by searching publication databases (Google Scholar, Semantic Scholar), relevant references, and related works.

### Data extraction

The following data were extracted from eligible studies: authors, year of publication, study design, follow-up, fusion type/instrumentation, sample size, and patient age. The primary outcomes of interest were the incidence of ASDeg and ASDis. ASDeg was commonly defined as alterations observed on magnetic resonance imaging (MRI) or plain radiographs at levels adjacent to the index level [5]. ASDis was defined by the presence of persistent back pain and new-onset radiculopathy or any other fusion-related pathologies that necessitated reoperation [5]. The selected studies are presented in the context of one or multiple iatrogenic alterations. The subsections for each iatrogenic alteration are structured as follows: introduction, description of iatrogenic alteration, explanation of potential pathomechanism leading to ASDeg, presentation of review results (state of the literature), discussion, and key takeaways. The review concludes with a classification of the iatrogenic changes presented based on the available evidence.

## Muscle damage

This section illustrates the potential effect of iatrogenic muscle damage on the biomechanics of the spine up to the development of ASDis or ASDeg. The spinal muscles are an important component of the stabilization system of the spine [18]. They fulfill two crucial functions: First, they provide passive support similar to the ligaments due to their non-active tissue consisting of muscle fasciae and tendons [19]. Second, and more importantly, they provide active stability by controlling and limiting motion [20]. This active stabilization takes place during normal daily activities, where forces applied by the muscles relieve the passive tissue, but on the other hand, it also takes place in traumatic situations, in which excessive stretching of the passive structures is prevented by opposing muscle reflexes [21]. Since the stability of the spine results from the complex interaction of all trunk muscles [22], the contributions of the individual muscles depend strongly on their position (lever arm, direction of force) in certain movements and the muscle’s physiological ability to generate a particular amount of force [20]. The back is stabilized by different muscles. The intrinsic back muscles run along the thoracolumbar spine and attach caudally to the iliac wing, sacroiliac joint, and sacrum [23]. The erector spinae muscles (iliocostalis, longissimus, spinalis) are the primary extensors of the spine and ensure an erect posture. But they also facilitate a controlled flexion and extension and contribute to lateral bending when unilaterally activated [24]. As part of the medial track of the erector spinae muscles, the multifidi muscles with short but thick muscle fibers span one (deep layers) to four (superficial layers) segments of the spine. With their large physiological cross-sectional area (PCSA) they are thought to be a strong stabilizer during flexion, extension, and lateral bending of the lumbar spine [25, 26]. Rather than stability, the short intersegmental muscles (interspinalis, intertransversarii, short rotator) are assumed to provide proprioceptive feedback [27]. The abdominal muscles act as antagonists to the back muscles and play an important role in flexion and axial rotation [28].

### Iatrogenic alteration

**Fig. 3:**
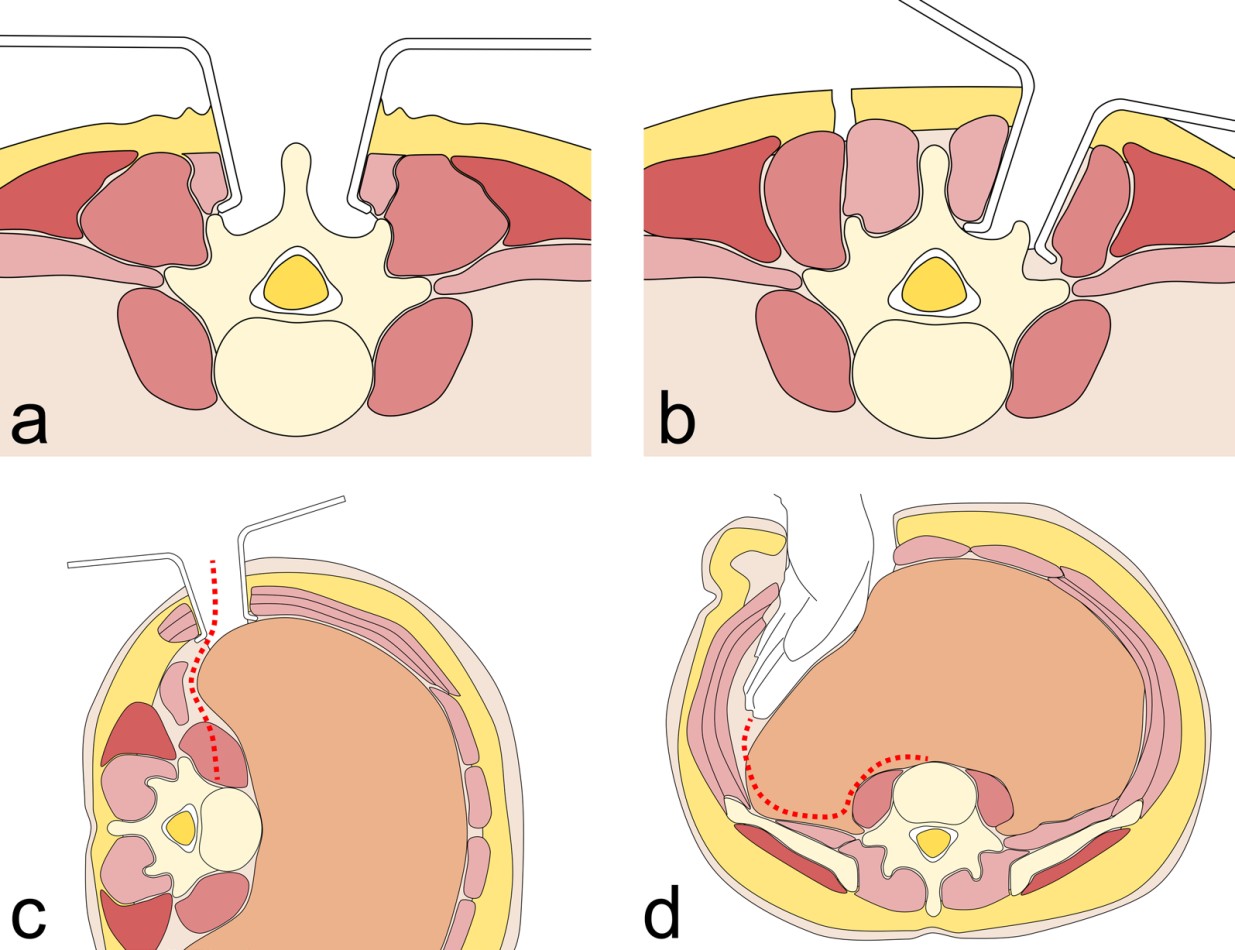
**a** Open midline approach: dissection of sacrospinalis down to the spinous process, **b** Wiltse approach: with dissection of multifidus and longissimus, **c** Retroperitoneal approach: sweeping of intraperitoneal contents down to psoas muscle, **d** Transpsoas approach: dissection of psoas muscle and peritoneum and splitting of psoas muscle. Adapted from AO Surgery Reference, https://surgeryreference.aofoundation.org, with permission

In open surgery, iatrogenic damage to the muscles cannot be prevented. The damage caused by the surgical incision with a scalpel or electro-cutter and the retraction can lead to postoperative muscle fibrosis, muscle atrophy, or fatty infiltration [29, 30]. However, the severity and the locality of these pathologies strongly depend on the type of incision and the performed surgery. The open midline approach (Fig. 3a) dissects and detaches the posterior fasciae and muscles (multifidus, longissimus, iliocostalis, and intertransverse muscles) along the spinous processes and lamina. In the paraspinal interfascial or Wiltse approach (Fig. 3b), the lumbar dorsal fascia is pierced lateral to the midline, followed by blunt dissection of the medial multifidus and lateral longissimus [31]. This paraspinal interfascial approach is often performed as a minimally invasive surgery (MIS) sparing the integrity of the muscles to a large extend [32, 33]. Anterior approaches (Fig. 3c) provide adequate access to the entire ventral surface. Incision and access usually include a midline or paramedian incision with a retroperitoneal corridor and vascular mobilization and dissection [34]. Muscles that are potentially affected include rectus and transversus abdominis, internal oblique, and psoas major. The transpsoas approach (Fig. 3d), bluntly dissects peritoneum and psoas muscle, which might be split to grant lateral access to the operated levels. Both anterior and lateral approaches are often combined with percutaneous pedicle screw placement that limits but cannot completely avoid dorsal muscle damage. In summary, each approach results in some damage to the spinal muscles, but the affected muscles and the extend depends on the surgical approach.

### Pathomechanism

When active or passive muscle stabilization is reduced due to the consequences of muscle injuries of the surgical approach, the load must be compensated by other structures, e.g., the IVD or the paraspinal ligaments. Punjabi therefore hypothesized that injury and degeneration to the musculotendinous complex is a major cause for lower back pain [18, 35]. While damage to the erector spinae muscles through posterior approaches is likely to affect the inclination (flexion) stability, damage to the abdominal muscles from anterior approaches could result in limited reclination (extension) stability. As both muscle groups are multisegmental structures, reduced integrity is unlikely to have only a local effect on the adjacent segment of the fusion, but rather on the global stability of the spine. At best, it would be conceivable that the global increase in stress contributes to accelerated degeneration first manifesting in the adjacent segment. The multisegmental nature however does not apply to the deeper layers of multifidus muscle, which consists of a multitude of unisegmental units. Since the multifidus is a muscle that is thought to stabilize flexion, shear or rotation [25, 26], an injury might therefore have a severe impact on adjacent segment stability. Furthermore, muscle damage potentially compromises the ligamento-muscular protective reflexes [21, 36].

### State of the literature

Respective damage to the muscles depends on the direction of the surgical approach (posterior, posterolateral, lateral, or anterior) and the technique (open midline, minimally invasive, endoscopic). Table 1 summarizes various studies that show the extent of the muscles’ cross-sectional area (CSA) reduction caused by a specific surgical approach:

- Within 1 year after open midline approach for posterior lumbar interbody fusion (PLIF), a retrospective study reported the multifidus to lose up to 40% of its CSA on MRI [29].
- A prospective randomized study revealed a 27% decrease in CSA of the erector spinae muscles distal but only slight changes proximal to arthrodesis after posterior open midline approach at 6-month follow-up [37].

**Table 1:** Reduction in cross-sectional area (CSA) after different surgical approaches

Author	Year	Study type	Approach	Follow-up (m)	Multifidus CSA reduction	Erector spinae (longissimus) CSA reduction
Cho [29]	2020	Retro	Open midline	12	up to 40%	–
Gille [37]	2007	Pro	Open midline	6	–	Proximal: 4% Distal: 27%
Dave [38]	2022	Pro	Open midline MIS	12	45.5 ± 12.4% 25.8 ± 9.6%	–
Hyun [39]	2007	Retro	Open midline MIS (PI)	6–18	20.7% 4.8%	–
Putzier [40]	2016	RCT	Open midline MIS (PI)	12	Index: 56.8%* Adjacent: 1.6% Index: 3.0% Adjacent: 1.8%	Index: 40.7% Adjacent: -2.8% Index: 33.5% Adjacent: 1.3%
Fan [41]	2010	Pro	Open midline MIS (PI)	14	Index: 36.8% Adjacent: 29.3% Index: 12.0% Adjacent: 8.5%	–
Kim [42]	2005	Retro	Open midline MIS (perc.)	20.6 21.5	30.3% 3.7%	–

*Pro* prospective, *Retro* retrospective, *CSA* cross-sectional area, *MIS* minimally invasive surgery, *PI* paramedian interfascial, *perc*. percutaneous

^*^volume reduction

Several prospective clinical studies compared open versus MIS approaches and report the postoperative changes in posterior muscles:

- Patients undergoing a primary single-level (L4-5 or L5-S1) transforaminal lumbar interbody fusion (TLIF) showed a significant difference in mean CSA-reduction of the multifidus when operated with the open midline approach (45.52 ± 12.36%) compared to MIS (25.83 ± 9.64%) [38].
- Comparing the paramedian interfascial approach for TLIF and the open bilateral midline approach for PLIF after 12 months at index level revealed a 54.2% volume reduction and increased relative fat content of multifidus, while no significant differences were observed for the longissimus [40]. At the adjacent level both multifidus and longissimus showed no significant changes in muscle volume and relative fat content.
- Direct comparison of MIS (paramedian interfascial approach) and open midline approach for PLIF in 59 patients showed a mean reduction of multifidus CSA of 12.2% at the operative level (8.5% non-operative) in the MIS group compared to 36.8% (29.3%) in the open midline group more than 1 year postoperatively [41].
- A retrospective case study found that MIS (paramedian interfascial approach) limited postoperative muscle atrophy to 4.8% compared to a volume loss of 20.7% with the open midline approach [39].

Several clinical studies compared open versus MIS approaches and reported the impact on muscle performance and inflammatory reaction:

- A comparison of muscle activation patterns 1 day preoperatively and 1 month postoperatively in 19 MIS TLIF patients revealed compensatory muscle coordination patterns and decreased paraspinal muscle activities during forward reaching compared to a control group [43].
- Based on a retrospective case study, patients who underwent MIS (percutaneous pedicle screw placement) showed less paraspinal muscle damage than those with an open midline approach for pedicle screw fixation while having improved postoperative trunk muscle performance [42].
- An analysis of inflammatory cytokines in 20 patients suggests that MIS (paramedian interfascial approach) may reduce muscle injury and systemic inflammatory reactions during the early postoperative recovery compared to the open midline approach [33].

There is clear evidence in the literature that muscles at the operated level lose cross-sectional area. Furthermore, the performance comparisons of MIS approaches with open surgery suggest that the extent of iatrogenic damage is crucial for the long-term outcome. Although fusion-surgery-induced muscle damage is evident, clinical studies on the potential effect of muscle damage on the development of ASDis are scarce. Nevertheless, the effect of muscle damage on ASD can be indirectly analyzed by comparing ASD outcomes of MIS procedures with those of open surgery. As shown in Table 2 and Appendix Table 13, the literature search revealed 18 studies, of which three studies compared open approaches and MIS for PLIF, nine for TLIF, four for mixed PLIF and TLIF, one for ALIF, and one compared open midline PLIF with MIS TLIF:

- A prospective study compared the MIS (paraspinal interfascial approach) and the open midline approach for one-level PLIF among 101 patients over a 7–9-year follow-up period. The MIS PLIF group showed significantly lower rates of adjacent segment degeneration and intractable back pain compared to the open midline PLIF group [44].
- In a retrospective study involving 100 patients who underwent single-level PLIF, MIS (paramedian interfascial approach) was compared to the open midline approach over an average follow-up of more than 8 years. The MIS group exhibited a significantly lower incidence of ASDis with a predicted disease-free survival rate of 98.5% at 5 years and 93.7% at 10 years, while the open approach was associated with a 3.97 times higher risk of developing ASDis [45].
- A retrospective study comparing MIS TLIF and open midline PLIF in 70 patients with a minimum 5-year follow-up, MIS TLIF had a significantly lower incidence of ASDeg compared to PLIF with an open midline approach [46].
- A retrospective case series with a minimum 5-year follow-up compared 121 patients undergoing TLIF procedures with either MIS or an open midline approach. Both surgical approaches had similar clinical outcomes, but MIS showed a significantly lower incidence of ASDeg with a rate of 33.3% compared to 59.4% for the open midline approach [47].
- In a retrospective cohort study of 68 patients who underwent TLIF with at least 6 months of postoperative follow-up, the study compared the risk of ASDis between open approaches and MIS. Although there was a trend towards a decreased risk of ASDis in the MIS group, the difference was not statistically significant [48].
- Another study compared 5-year outcomes of 60 patients undergoing either MIS or an open midline approach for TLIF. It concluded that MIS is comparable to an open approach in terms of long-term outcomes, interbody fusion rate, and prevalence of adjacent segment degeneration [49].
- A second study comparing endoscopic MIS and an open midline approach for TLIF in 100 patients found that both groups had similar rates of adjacent segment degeneration and disease at 24-month follow-up [50].
- In a retrospective study comparing MIS and an open midline approach for TLIF in 83 patients over a 10-year period, both approaches showed comparable outcomes at 10 years, including radiographic fusion rates and the prevalence of secondary surgery for adjacent segment disease [51].
- A retrospective chart review, conducted over a minimum 10-year follow-up period, investigated 87 lumbar fusion patients undergoing either an open midline approach or MIS (percutaneous pedicle screw placement). Both surgical approaches showed similar incidence rates of adjacent segment disease (ASDis) at 33.3% [52].
- Among 53 patients undergoing anterior lumbar interbody fusion (ALIF) with less than 4 years follow-up, no differences in ASDis rates were observed in the comparison of the open midline approach and MIS (percutaneous) for pedicle screw placement [53].
- A retrospective 2-year follow-up study compared MIS (paramedian interfascial approach) and open midline approaches for PLIF. Reoperation due to adjacent segment pathology was found to be more common in the open midline group, with 14.6% (15 of 103 patients) requiring reoperation, compared to 5.8% (6 of 103 patients) in the MIS group [54].
- A retrospective study investigated MIS TLIF versus TLIF with an open midline approach in 80 patients over 60 months, finding an equal ASDis incidence of 10% in both groups [55].
- Another study compared the same techniques in 148 patients over 24 months, reporting ASDis rates of 0% for MIS TLIF and 1% for TLIF an open approach [56].
- A comparison of MIS and open midline procedures in 49 patients over 26 months revealed ASDis incidences of 4% and 8%, respectively [57].
- A separate study of 237 patients over 24 months found ASDis rates of 5.8% for MIS TLIF and 15.4% for TLIF with an open midline approach [58].
- A larger study followed 697 patients for over 60 months, reporting ASDis incidences of 5.9% and 11.6% in patients undergoing MIS and an open midline approach, respectively [59].
- A retrospective study of 206 patients undergoing MIS PLIF (paramedian interfascial) or PLIF with an open midline approach over 24 months found ASDis rates of 5.8% and 14.6%, respectively [54].
- Another study followed 57 patients for at least 3 years, with 84.2% undergoing lumbar fusion surgery with an open midline approach. Among these, 12.3% developed ASDis requiring further surgery, while none of the patients who received MIS (percutaneous pedicle screw placement) experienced symptomatic ASDeg or needed additional procedures [60].

**Table 2:** Differences in ASDeg and ASDis incidences between open and minimally invasive surgery (summary of Appendix Table 13)

	ASDeg incidence	ASDis incidence
Significantly higher in MIS group	–	–
Higher in MIS group (not significant)	–	1 [51]
No difference between MIS and open group	2 [49, 52]	4 [52–56]
Higher in open group (not significant)	2 [48, 50]	2 [57, 59]
Significantly higher in open group	3 [44, 46, 47]	4 [45, 54, 58, 60]

Retrospective studies comparing ventral and lateral fusion approaches in terms of the development of ASDeg can help to determine the impact of muscle damage, as ALIF with percutaneous pedicle screws limits the damage to the major dorsal muscles (Table 3, Appendix Table 14):

- A case study of 48 patients followed for at least 2 years, compared the outcomes of percutaneously instrumented ALIF and PLIF with open midline approach for lumbar spondylolisthesis treatment, finding that ALIF had a lower incidence of ASDeg (44.0% vs. 82.6%), but similar Japanese Orthopaedic Association (JOA) score and recovery rates [61].
- Analysis of 82 patients found that ALIF and lateral lumbar interbody fusion (LLIF) with minimally-invasive (percutaneous) pedicle screw placement are more effective than open midline PLIF in preventing ASDis and improving disk and foraminal height and lordosis, while all three techniques produced similar clinical outcomes in terms of visual analog scale (VAS) and Oswestry Disability Index (ODI) [62]. Reported incidences of ASDis were 37.0% in the ALIF group, 41.7% in the LLIF group, and 64.5% in the PLIF group based on an average of 35 months radiographic follow-up.
- A study on ALIF without posterior pedicle screw placement showed a significantly lower ASDeg rate of 13% as compared to 38% in patients undergoing PLIF with an open midline approach. The patients with a mean age of 55 were followed for more than 3 years [63].

**Table 3:** Differences in ASDeg and ASDis incidences between ventral and dorsal surgical approaches (summary of Appendix Table 14)

	ASDeg incidence	ASDis incidence
Significantly higher in ventral group	–	–
Higher in ventral group (not significant)	2 [64, 65]	–
No difference between ventral and dorsal group	1 [66]	4 [61, 64–67]
Higher in dorsal group (not siginifcant)	1 [68]	1 [62] (LLIF vs PLIF)
Significantly higher in dorsal group	2 [61, 63]	1 [62] (ALIF vs PLIF)

Although, not statistically significant, multiple retrospective studies found a trend in reduced ASDeg rates for ventral approaches when compared to dorsal techniques (Table 3):

- A comparison of 30 ALIF patients (percutaneous pedicle screw placement) and 40 LLIF patients (open midline approach) showed similar rates of ASDeg (53.3% ALIF vs. 47.5% TLIF) and ASDis 5-year survival rates (0.68 ALIF vs. 0.35 TLIF) [64].
- A recent study compared oblique lumbar interbody fusion (OLIF) with percutaneous pedicle screw placement to an open midline approach in 72 TLIF patients followed for 3 to 4 years. No significant differences in the occurrence of ASDeg (19.4% OLIF vs. 27.8% TLIF) or ASDis (2.8% OLIF vs. 5.6% TLIF) were observed [66].
- Another comparison of MIS OLIF (percutaneous pedicle screw placement) and TLIF with an open midline approach showed only insignificant differences in terms of ASDis (7.6% OLIF vs. 10% TLIF) [67].
- Three groups of patients (ALIF, TLIF, PLIF) followed for less than 2 years were compared in terms ASDeg occurrence. Although statistically insignificant, highest rates were found in the TLIF (24%) and PLIF (13%), with the lowest occurrence in the ALIF group (8%) [68].
- Comparing 103 patients with a minimum follow-up of 3 years showed ASDeg rates of 13% in the MIS ALIF group compared to only 4% in the MIS TLIF group [65].

### Discussion

There is clear evidence in the literature that surgical access leads to iatrogenic muscle damage and loss of cross-sectional area. Furthermore, literature shows that muscle damage is more extensive in open midline approaches than in MIS or endoscopic approaches due to smaller incisions, but also other factors like the extent of retraction [69, 70]. Therefore, MIS and endoscopic procedures offer clinical advantages in terms of preserving muscle volume and CSA, which appear to affect long-term performance and clinical outcomes. Accordingly, studies that examined the relationship between muscle damage and ASDeg showed a trend toward lower ASDeg rates for muscle-sparing approaches. This was shown in studies comparing minimally invasive techniques with conventional open approaches (Table 2) as well as in studies investigating differences between dorsal and ventral surgical access (Table 3) to the spine. However, the differences were not always statistically significant, most likely due to limited sample sizes within each study. Further, the tables do not differentiate between minimally invasive surgery (often referring to paramedian interfascial approach) and percutaneous pedicle screw placement.

Although the studies describe a link between muscle-sparing techniques and reduced ASDeg incidences, these observations alone are not conclusive of ASD. It should be noted that differences between anterior and posterior approaches are not limited to muscle damage but are associated with various other factors. For example, ligament damage or the achievable lordosis differs between dorsal and ventral approaches [71].

Moreover, the fact that MIS did not always significantly reduce ASDeg incidences may challenge an extensive effect of muscle damage on the development of ASDeg since varying degrees of muscle damage are apparent between the two approaches. Although muscle damage is limited to the fusion level and adjacent segments, there appear to be global effects on spinal stability and loading. In silico studies suggest increased forces in the spinal column if muscle insufficiencies are simulated [72–74]. It cannot be ruled out that monosegmental muscle damage in combination with other factors, such as a rigid adjacent segment, may have a reinforcing effect. However, the biomechanical mechanism by which this reinforcement occurs specifically in the adjacent segment of fusion is probably rather small and remains somewhat unclear.

Based on current literature it can be concluded that iatrogenic muscle damage plays a role in the development of ASDis, but it is not the sole accelerator. Regarding clinical practice, muscle-sparing techniques are preferable but not always feasible in complex cases where extensive decompression or challenging cage insertion is needed. Here, early postoperative training and therapeutic measures should be considered to mitigate adverse effects of iatrogenic muscle damage [43].

### Key takeaways

1. *Muscle is an important passive and active stabilizer of the spine. *In silico* studies suggest an increased global force in the spine due to muscle insufficiencies.*
2. *There is clear evidence in the literature that the surgical approach leads to long-term damage to the musculature. The extent of damage depends on the size and type of surgical access. MIS or endoscopic approaches can minimize this damage.*
3. *Clinical evidence suggests that anterior or lateral approaches reduce the risk of developing ASDis, but this cannot be attributed solely to sparing of the back muscles, as also other factors differ between the two approaches.*
4. *Although not always statistically significant, minimally invasive muscle sparing techniques have shown superiority to conventional spinal fusion approaches regarding ASDeg-outcomes. This might indicate that muscle damage plays a relevant role in the development of ASDeg.*
5. *Iatrogenically induced damage to the musculature may lead to a deterioration of the force conditions in the spine, which could accelerate degenerative effects in the spine on a global level. Isolated influences on the adjacent segment, on the other hand, appear less pronounced.*

## Ligament damage

This section illustrates the potential effect of iatrogenic ligament damage on the biomechanics of the spine up to the development of ASDeg. As part of the passive stabilization complex, the ligaments consist of highly structured collagen fibers to optimally withstand tensile forces. Foremost, they limit the range of motion (ROM) and protect the spinal canal, but they are also assumed to act as transducers that provide information about the position and motion of vertebrae [18]. Whether a ligament is loaded depends on its anatomical position and the respective motion pattern. In contrast to the other ligaments, the intervertebral ligamentum flavum (LF) is pre-tensed and elastic to avoid buckling into the spinal canal. Together with the posterior longitudinal ligament (PLL) it restricts flexion, while the opposing anterior longitudinal ligament (ALL) limits extension movements [75]. Interspinous and supraspinous ligaments (ISL & SSL) are activated during flexion. The intertransverse ligaments (ITL) may restrict lateral flexion [24, 75] but its influence is minimal, suggesting a possible proprioceptive role rather than a mechanical one [75].

### Iatrogeneic alteration

**Fig. 4:**
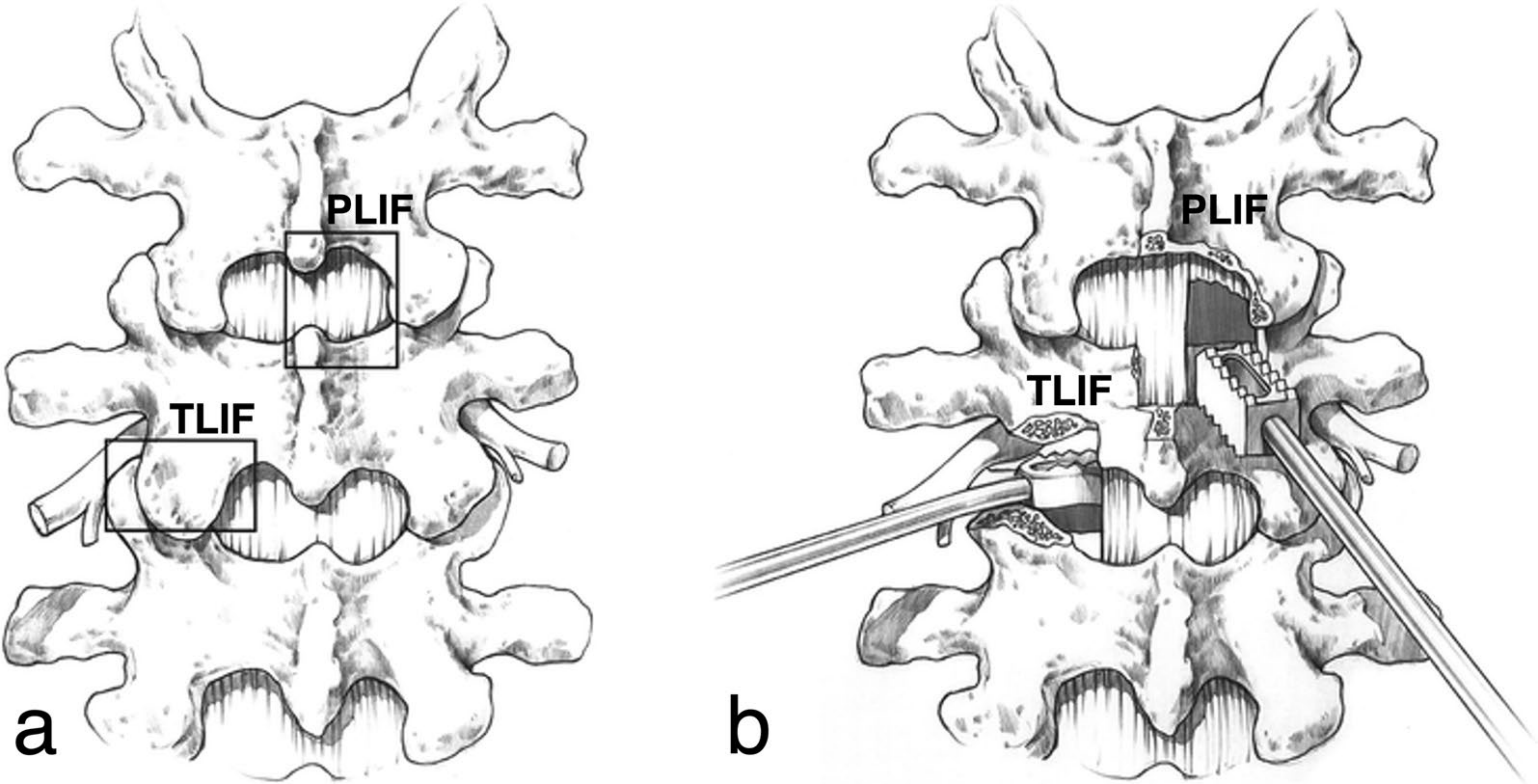
**a** Schematic representation of the posterior lumbar spine with access routes for PLIF on the cranial right and TLIF on the caudal left, **b** Cage insertion positions for PLIF on the cranial right and for TLIF on the caudal left Adapted from Cole et al. (2009) [76], distributed under the CC BY-NC 2.0 License (https://creativecommons.org/licenses/by-nc/2.0)

**Fig. 5:**
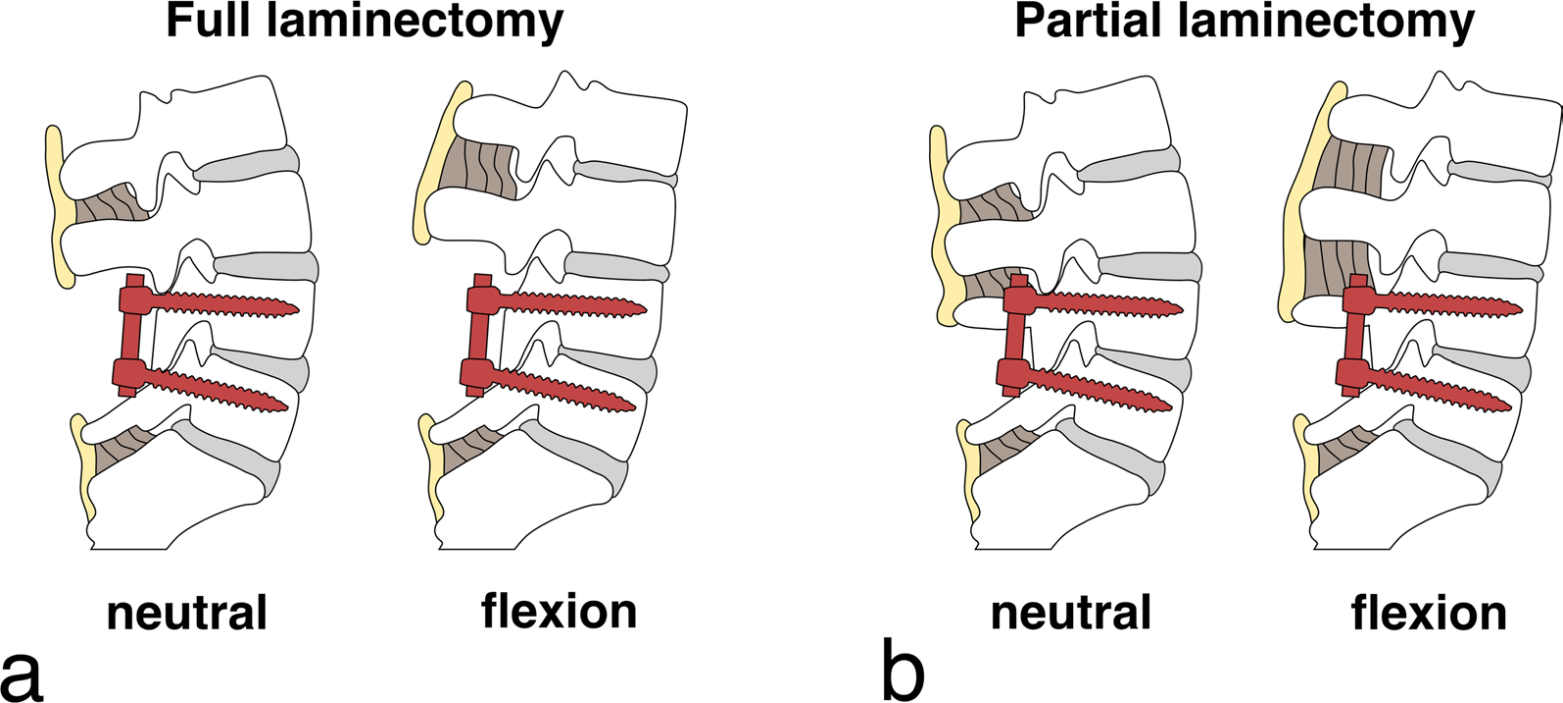
Instrumented spine at L4–L5 in neutral and flexion positions with **a** full laminectomy: complete removal of L4 spinous process and L3–L4 supraspinous and interspinous ligaments. No tension band mechanism between L3 and L4 spinous processes in flexion, **b** partial laminectomy: preservation of upper L4 spinous process, maintaining intact L3–L4 supraspinous and interspinous ligaments

Ligament damage is a largely unavoidable side effect of spinal fusion surgery. Which ligaments are affected depends on the surgical approach, and the extent and type of decompression performed. PLIF accesses the disc space by (unilateral) partial removal of the cranial and caudal spinous processes and laminae (laminotomy), including portions of the facet joint [76] (Fig. 4). This may result in complete resection of the SSL, ISL, and LF at the index level. To preserve the posterior ligamentous complex [76], the dorsal third of the ISL and the SSL may be spared. After retraction of the dural sac and nerve roots to the midline, the posterior annulus is exposed by (partial) removal of the PLL. For TLIF the disc space is accessed by a unilateral laminotomy and medial or full resection of the facet joint (Fig. 4). The extent depends on the necessary exposure and the side is chosen based on the patient’s symptoms or abnormalities [77]. This access reduces the retraction of nerve roots and mainly spares the posterior ligamentous complex, only necessitating partial removal of the LF and PLL to access the disc space. Both approaches are complemented by discectomy, cage insertion, and intervertebral and optionally posterolateral bone graft placement. Independent of the approach, cases of severe spinal stenosis can necessitate a decompression by full laminectomy, thus, removal of the spinous process and dissection of the cranially and caudally attached ligaments (SSL & ISL) and muscle attachment points (Fig. 5a). During ALIF the posterior ligaments stay untouched as accessing the disc space only demands resection of the ALL.

### Pathomechanism

Resection of short, monosegmental ligaments at the index level should have a negligible effect on the adjacent segments because the index level is immobilized by the fusion. However, in the case of larger decompressions, ligaments in the adjacent segment are also affected, for example in a full laminectomy (removal of the whole spinous process, Fig. 5a). Missing ligaments that contributed to the passive stability in flexion directly affect flexion stability in the adjacent segment and may contribute to increased flexion ROM. Therefore, the facet joints and the posterior IVD are subject to increased tensional forces, while the anterior part of the IVD may experience excessive compression. In addition, damage to the ligamentous complex may also compromise shear and rotational stability, potentially leading to hyper-rotation and hyper-shear straining the IVD.

Apart from iatrogenic damage, a so-called subfailure injury can occur when the ligaments are stretched beyond their physiological limits, but before reaching their failure point [78]. Punjabi hypothesized that these subfailures damage the mechanoreceptors, corrupting the muscle response pattern, and eventually leading to overloading of the spinal segments (disc, facet joints, ligaments) [79]. Even environmental changes without direct ligament damage seem to affect the ligamentous structures. For instance, indirect decompression through ALIF may lead to remodeling of the ligamentum flavum [80]. So light irritation, altered mechanical loading, or environmental changes themselves may trigger a degenerative (and symptomatic) response of the ligaments.

Another point that is perhaps greatly neglected in today's literature is the multisegmental effect of ligaments. The SSL, for example, spans multiple segments and runs along the spine as a rope-like structure to prevent global hyperflexion of the spine. Resection of the ligament, or even a slack SSL from a fused segment, might result in a reduction of this protective function. Slackening of the SSL and ISL potentially occurs with every spinal fusion and may lead to an overload of the adjacent segment, as overstretching is no longer prevented in strongly flexed positions.

### State of the literature

The literature can be divided into studies investigating ASD after full laminectomy at the proximal fixed vertebra (Fig. 5a) or decompression at the segment adjacent to fusion. The former was the subject of four studies (Table 4, Appendix Table 16):

- A prospective study with more than 5 years of follow-up observed four- to almost six-time higher occurrences of ASDeg in patients undergoing a full laminectomy (including resection of the cranial laminae, spinous process, ISL, and SSL) when compared to patients that had a facet joint resection or hemilaminectomy, respectively [81].
- A prospective randomized study investigating the long-term effects (12.6 years follow-up) of lumbar fusion for isthmic spondylolisthesis [82], found that almost all patients that developed ASDeg, had PLF combined with removal of the loose lamina (laminectomy).
- A retrospective analysis of 71 fusion patients revealed a significantly larger ASDeg incidence (49%) in patients with a full laminectomy at the proximal fused segment than in patients without (23%) [83].
- A retrospective analysis of 378 patients with spondylolisthesis who underwent L4-5 fusion surgery revealed that, in comparison to subtotal laminectomy, a full laminectomy resulted in a higher incidence of reoperation at the adjacent level due to ASDis (5.2% vs 19.8%) [84].

**Table 4:** Differences in ASDeg and ASDis incidences between index segment decompression and no or only partial index segment decompression (summary of Appendix Table 16)

	ASDeg incidence	ASDis incidence
Significantly higher in no/partial decompression group	–	–
Higher in no/partial decompression group (not significant)	–	–
No difference between groups	–	–
Higher in index segment decompression group (not significant)	–	–
Significantly higher in index segment decompression group	3 [81–83]	1 [84]

Seven retrospective studies looked at the occurrence of ASDeg in fusion surgeries with decompression of the adjacent segment (Table 5, Appendix Table 15):

- Decompression in the adjacent segment to PLF/PLIF fusion was found to increase the ASDis risk (30%) when compared to fusions without adjacent segment decompression (20%) [85].
- A similar trend was observed in a cohort of 154 patients with circumferential fusion or PLF. The rate of ASDeg for adjacent segment decompression was 30% compared to 8% in the no-compression group after an average follow-up of 29 months [86].
- A small cohort study investigated 25 segments with and 15 without adjacent segment decompression [87]. The incidence for ASDeg (64%) and ASDis (36%) was significantly higher with adjacent segment decompression than without (ASDeg 20%, ASDis 6.7%). Average follow-up was 53 months.
- An evaluation of 912 patients showed that a fusion combined with an additional laminectomy in the adjacent segment increased the ASDis risk by a factor of 2.4 [88].
- Patients who had a laminectomy before AxiaLIF fusion surgery were reported to have a higher incidence of ASDis (57% vs. 11.3%) [89].
- In a cohort of 54 patients who underwent L4-5 PLIF for L4 degenerative spondylolisthesis, patients with simultaneous decompression at L3-4 exhibited significantly higher ASDeg rates (p = 0.009) [90]. However, ASDis rates did not differ significantly between PLIF only and additional L3-4 decompression patients. At a mean follow-up of 55 (24 – 148) months, the overall ASDeg and ASDis incidences were 57.4% and 13.0%, respectively.
- A study of 161 patients with a short follow-up of less than a year showed no significant difference in terms of the development of new ASDeg between instrumented fusions with or without laminectomy in the adjacent segment (p = 0.36) [91].

**Table 5:** Differences in ASDeg and ASDis incidences between adjacent segment (AS) decompression and no AS decompression (summary of Appendix Table 15)

	ASDeg incidence	ASDis incidence
Significantly higher in no AS decompression group	–	–
Higher in no AS decompression group (not significant)	–	–
No difference between groups	1 [91]	–
Higher in AS decompression group (not significant)	–	1 [90]
Significantly higher in AS decompression group	2 [87, 90]	5 [85–89]

As indicated earlier in cases without midline decompression, PLIF is still accompanied by a (partial) resection of the SSL, ISL, LF, and PLL incision at the index level (Fig. 4). Anterior, and lateral approaches (e.g., ALIF, LLIF) combined with percutaneous pedicle screw placement leave posterior ligaments intact. If ligament damage is a causative factor, it is expected that anterior and lateral approaches will result in a lower incidence of ASDis compared to posterior approaches. As presented in Table 3 and Appendix Table 14, the literature comparing ventral and dorsal approaches reported a tendency toward lower ASD incidences for ventral approaches. Nevertheless, an influence of the extent of muscle impairment cannot be ruled out as posterior approaches always induce both muscle and ligament damage. Further, differences could be also attributed to other factors like segmental lordosis restoration. Given the same surgical approach (open or MIS) a comparison of PLIF with TLIF could minimize this bias since TLIF is an approach with similar muscle damage and restoration quality but that spares the spinous process and parts of the intervertebral and posterior ligaments. Except for one retrospective study with short follow-up and small patient sample [68], the review revealed no studies that directly compared open PLIF and open TLIF or MIS-PLIF and MIS-TLIF approaches in terms of ASDeg incidence.

### Discussion

Despite the importance of the intact posterior ligamentous complex for spinal stability [92], research on the potential effects of ligament damage on the development of ASDeg is still scarce. A biomechanical study proved that midline decompression without fusion significantly increases segmental ROM (up to 20%) suggesting the importance of posterior ligaments [93]. A retrospective study showed that in patients with preserved adjacent posterior complex fewer (6,5% vs. 24,3%) patients develop instability at the adjacent cranial segment [94]. The caudal adjacent motion segment showed a similar trend, where no patient with preserved integrity developed adjacent instability, compared to 5.6% without preserved integrity. Consistently, the presented clinical studies revealed that damaging the integrity of the posterior complex between the fused segments and the neighboring motion segments seems to jeopardize lumbar spine stability and increase the risk for the development of ASDeg.

In particular, the prospective study by Liu et al. [81] should be highlighted in this context. They evaluated three groups of patients who underwent PLIF but with varying extent of posterior decompression. Although all patients were operated with the same open midline approach, facet joint resection and semilaminectomy resulted in an ASDeg incidence of only 12% and 17%, respectively, while 71% of patients undergoing complete laminectomy developed ASDeg within 5 to 7 years. Additionally, reoperation for ASDis occurred only in patients who underwent complete laminectomy. The results underscore the protective role of an intact posterior complex and suggest that even partial preservation of posterior structures may significantly reduce the risk of ASDeg and ASDis.

Furthermore, the comparison between anterior/lateral and posterior approaches, indicates a reduced ASDeg rate with posterior complex sparing approaches. Nevertheless, ASDeg is also observed in anterior approaches, albeit to a lesser extent. This effect could be attributed to reduced muscle damage (see Muscle Damage) or to natural degeneration that would also develop without spinal fusion. On the other hand, the above-described leveraging of the multisegmental protective effect could be a decisive point. Ekman et al. already hypothesized that a disruption of the posterior tension band function could cause instability and accelerate degeneration [82]. In particular, the multisegmental SSL could play an important role, which has been insufficiently investigated to date. As an example, a study on multilevel cadaveric spine segments showed that the detachment of the SSL leads to a significant increase of the ROM in adjacent segments [95]. Further biomechanical and clinical investigation of this effect will be important to understand the causality between disruption of multisegmental passive structures and ASDeg. In summary, the literature suggests that iatrogenic ligament injury may be a critical factor in the development of ASDeg and surgeons are advised to be judicious in the extent of decompression in each case.

### Key takeaways

1. *Ligament damage is an unavoidable side effect of spinal fusion surgery. The LF, the PLL, and the posterior ISL and SSL are particularly affected. The degree of injury and the structures that are injured depend on the surgical approach and the type of decompression.*
2. *Ligament sparing spinal fusion techniques have been associated with lower risk for ASDeg development, but impact of concurrent muscle sparing cannot be differentiated.*
3. *Decompressions in the adjacent segment without fusing it are associated with a higher rate of ASDeg.*
4. *A comparison between anterior/lateral approaches without ligamentous injuries and posterior approaches with ligamentous injuries supports the observation that ligament preservation is important for the prevention of ASDeg.*
5. *Biomechanical evidence suggests that SSL and ISL rupture has an adverse effect on loading redistribution. The multisegmental effect of SSL, which has not been studied extensively, could be crucial in this respect.*

## Facet joint damage

This section illustrates the potential effect of iatrogenic facet joint damage on the biomechanics of the spine up to the development of ASDeg. The bilateral facet joints are diarthrodial synovial joints located in the posterolateral part of the spinal column connecting the inferior and superior articular processes of two adjacent vertebrae [96]. In contrast to the intervertebral disc, which provides a soft transmission of force, the segmental movement here is guided by bone-to-bone contact. The ligamentous joint capsule is richly innervated with mechanoreceptive, proprioceptive and nociceptive nerve endings [97, 98] that provide feedback to the central nervous system. Along with the IVDs, the facet joints ensure segmental stability by transferring loads and collecting sensory feedback. The facet joints’ contribution to spinal stability and load transfer was assessed in a series of cadaveric and computational experiments. In slight extension (2°) the facet joints were also found to transmit around 16% axial loading [99], and up to 25% together with the spinous processes during extensive extension [100, 101]. The transmission of forces in the axial direction through the facet joints has been somewhat overestimated in the older literature, due to studies that measured significant force transmission with force-measuring foils [102]. Recent studies no longer share that view [75] but claim that their main mechanical function is to guide and limit axial rotation and prevent extensive shear strains [103, 104]. Resection studies revealed that they contribute 49% to axial rotation stability and around 15% to lateral and anterior shear stability in the lumbar spine [75].

### Iatrogenic alteration

**Fig. 6:**
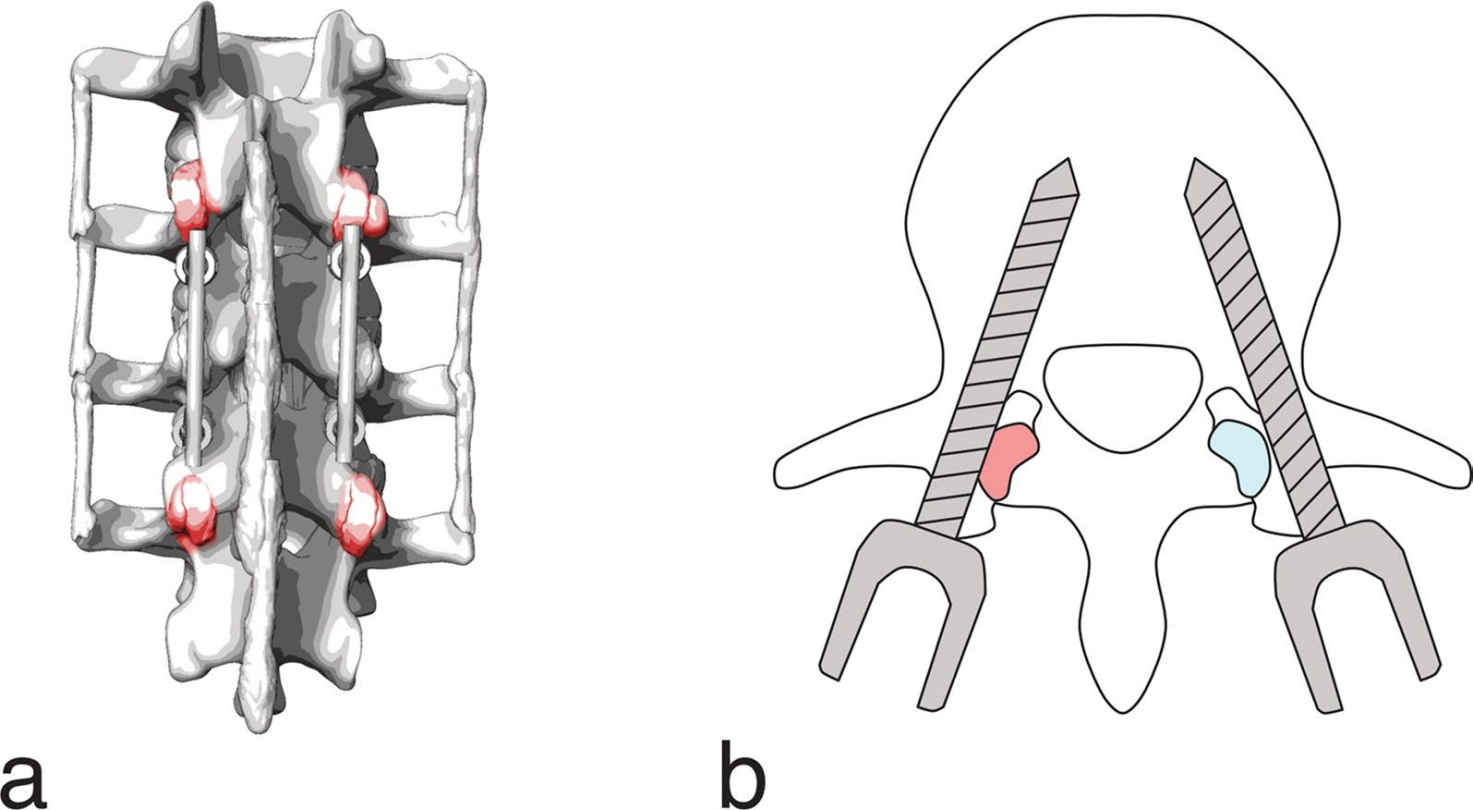
**a** Potential locations of facet joint violations on the cranial and caudal adjacent segment (posterior view) **b** Facet joint violation (red) by the left pedicle screw and no violation (blue) by the right pedicle screw

The major risk for the facet joints in the adjacent segment stems from the pedicle screw placement in the proximal fused vertebra. A violation of the superior facet joint is usually assessed on axial CT slices and occurs when the pedicle screw or the pedicle screw head lies within or touches (abutting) the facet joint (Fig. 6). The incidence of superior facet joint violations during pedicle screw placement was widely reported in the literature (Table 6). For open fusion approaches between 12% and 36% of patients are affected [53, 105–109]. Higher incidences of 28% to 50% were reported for minimally invasive approaches with percutaneous pedicle screw placement [53, 107, 109–111]. Bilateral superior facet joint violation occurred only in 1% to 14% of the patients [106, 109, 110], while the L4 and L5 levels were reported to be the most prone to superior facet joint violations. Depending on the symptomatology, fusion surgery may require a full or partial resection of the facet joints at the index level to make room for the nerve roots or to provide access to underlying structures, e.g., TLIF necessitates a unilateral facetectomy to reach the disc space (Fig. 4b).

**Table 6:** Incidence of superior facet joint violation in fusion surgery

Author	Year	Study type	Fusion + Instrumentation	Sample size	Superior facet joint violation *bilateral*	Inter-rater reliability
Moshirfar [106]	2006	Retro	PLF + OPS	204	24% *14%*	κ = 0.63 (Cohen)
Shah [108]	2003	Pro	PLIF/ALIF + OPS (Wiltse)	106	32–35%	κ = 0.88 (Cohen)
Chen [105]	2008	Pro	PLIF + OPS (Weinstein) PLIF + OPS (Roy-Camille)	96 20	33–36% 100%	NR
Park [110]	2011	Retro	LF + PPS	92	50% *12%*	single rater
Patel [107]	2020	Pro	TLIF + OPS TLIF + PPS	105 120	30% 41%	*r* = 0.98 (Pearson)
Teles [109]	2018	Retro	PLIF + OPS PLIF + PPS	81 50	12% *1%* 28% *12%*	κ = 0.789 (Cohen)
Babu [60]	2012	Retro	LF + OPS LF + PPS	252 306	28% 40%	*r* = 0.98 (Pearson)
Zhao [111]	2020	Retro	TLIF + PPS	91	34%	κ = 0.784 (Cohen)

*Pro* prospective,* Retro* retrospective,* PLIF* posterior lumbar interbody fusion,* PLF* posterolateral fusion,* ALIF* anterior lumbar interbody fusion,* LF* lumbar fusion,* TLIF* transforaminal lumbar interbody fusion,* OPS* open pedicle screw placement,* PPS* percutaneous pedicle screw placement,* NR* not reported

### Pathomechanism

As the fusion construct limits motion, facet joint damage or removal at the operated level does not compromise mechanical stability. However, a severe violation of the facet joints’ integrity in the adjacent unfused segment could impact their mechanical function to resist shear and rotational movements. Without fully functional facet joints, the shear stress and rotational stress on the disc and other functional tissues increases as they must compensate for this stress. Depending on the kind of loading, the excessive shear stresses could result in overextension of the disc or the enveloping ligaments due to compensatory load distribution to alternative structures. As a second consequence, iatrogenic injury to the non-fused facet joints in the connecting segment can lead to the progression of facet joint degeneration [112] accelerating ASDeg. Furthermore, screws and rods can block movement upon impact, potentially leading to impingement, mechanical irritation, fatigue fractures, or discomfort. Resulting inflammatory reactions can cause pathology of the adjacent level, as pain signals originate from the irritated nociceptive nerve endings of the facets [113]. Thirdly, damage or removal of the facets in either adjacent or index levels may disrupt the mechanical and proprioceptive feedback to the central nervous system, which is responsible for an adequate muscular response [18]. Consequently, faulty paraspinal muscle activity may trigger a cascade of instability, excessive stresses, and subsequent damage of the functional tissue in the adjacent segment.

### State of the literature

Several clinical studies have investigated the association between superior facet joint damage﻿ and ASDeg (Table 7, Appendix Table 17). Two retrospective studies looked at incidence of ASDeg:

- Analysis of 87 patients undergoing instrumented PLF or PLIF revealed higher ASDeg rates for patients with (55%) than without (42%) superior facet joint violations. The patients had a mean age of 65 years and were followed for over 13 years on average [114].
- A small cohort of 49 patients with more than 11 years of follow-up underwent ALIF with percutaneous pedicle-screw placement but showed no significant differences in ASDeg incidences with respect to superior facet joint violations [115].

A further three studies, investigated the occurrence of ASDis in the context of superior facet joint violations (Appendix Table 18):

- In a patient cohort of 630 patients undergoing posterior transpedicular fusion, 96 had superior facet joint violations [116]. The incidence of ASDis within this group was much higher (57%) compared to the non-violated group (13%). The mean age was 62 years and the average follow-up 52 months.
- Analysis of 237 open midline PLIF and TLIF patients revealed that a fourth of the patients with superior facet joint damage developed ASDis while patients with a preserved superior facet joint had a ASDis prevalence of only 1.6% [117]. The patients’ mean age was 54 and they were followed for about 2.5 years.
- A significant difference in the incidence of ASDis (19.6% vs. 9.4%) was observed between 112 patients with and 128 patients without superior facet joint injuries [118]. The patients with a mean age of about 64 years were followed for three years and underwent lumbar fusion surgery with posterior instrumentation.

**Table 7:** Differences in ASDeg and ASDis incidences in patients with or without facet joint violations (summary of Appendix Table 17)

	ASDeg incidence	ASDis incidence
Significantly higher in patients without facet joint violations	–	–
Higher in patients without facet joint violations (not significant)	1 [115]	–
No difference between groups	–	–
Higher in patients with facet joint violations (not significant)	1 [114]	–
Significantly higher in patients with facet joint violations	–	3 [116–118]

Disregarding the small cohort study with ALIF patients, the literature agrees that the integrity of facet joints of the superjacent segment appears to be linked to adjacent level pathologies. In addition, the following three studies compared pedicle-screw placement techniques in terms of ASDeg incidences:

- A prospective comparison of instrumentation configuration showed no significant differences between facet preserving and facet abutting pedicle screw insertion techniques in terms of ASDeg incidence [119].
- The second study investigating facet-preserving cortical bone trajectory reported lower ASDeg incidences (52%) of statistical significance when compared to traditional transpedicular trajectories (64%) [120].
- The third retrospective review showed significantly higher ASDeg rates for the facet-sparing cortical bone trajectory (42%) than for the traditional transpedicular trajectory (13%) [121].

However, their outcomes should be interpreted with caution since they do not report the true incidence of facet joint violations and only group by the techniques with a higher likelihood of superior facet joint damage.

### Discussion

Despite the potential association between intraoperative facet joint injuries and the development of ASDeg, there is a paucity of literature on this topic. The existing clinical studies agree that iatrogenic facet joint damage correlates with increased rates of ASDeg. Biomechanical studies support this observation by highlighting the facet joints’ contribution to segmental stability. A cadaveric experiment confirmed increased ROM in axial rotation (10–13%) in the adjacent segment after simulated bi-lateral superior facet joint violation [122]. Another biomechanical cadaver study suggests that blockage of the superior facets by pedicle screws alters the ROM of the adjacent motion segment in flexion–extension [123].

However, despite these observations, it is unlikely that the clinical rate of ASDeg can be explained by iatrogenic facet joint injury in the adjacent segment. That potentially destabilizing bilateral cephalad facet joint injury occurs in only 1–14% of cases [106, 109, 110], questions a strong mechanical linkage between facet joint violations and degenerative adjacent segment instability. Furthermore, the presence of ASDis in both cranial and caudal segments contradicts a purely mechanical explanation. Okuda et al. reported that 30% of ASDis occurred caudally [124]. However, the reviewed literature has not reported any instances of facet joint damage in the lower adjacent segments. Additionally, it should be noted that as degeneration progresses in the segment instability appears to decrease. This was shown for adjacent facet joint osteoarthritis [125], which seems to induce higher progression rates in segments with violated facet joints [112].

In terms of the joints' involvement in mechano- and proprioceptive feedback, porcine and feline models demonstrated that electrical stimulus of the facet joints stimulates muscle activity at the same level [126, 127]. Further, injection of physiologic saline into the facet joint affected nerve signaling between the intervertebral disc and the paraspinal musculature [128]. Nevertheless, there is a lack of research regarding whether indirect overloading of adjacent segments may occur due to a lack of proprioceptive signals in the fused segment. Since immobilized facet joints cannot provide proprioceptive feedback, this could affect the adjacent segment. However, this theory applies not only to the facet joint, but to all structures with proprioceptive feedback, such as ligaments or muscles. It seems more likely that the pathologies reported as ASDis are due to irritating implant contact, inflammatory reactions, or painful soft tissue impingement. Nevertheless, further research is needed to investigate the observed correlation between facet joint damage and ASDeg.

Overall, iatrogenic damage to the facet joints may accelerate the development of ASDeg, but it alone cannot explain the prevalence of ASDeg, especially in the subjacent levels. Iatrogenic damage to the facet joints is an additional factor contributing to the development of ASDeg, rather than an initiator of the degenerative cascade, as it is generally assumed that disc degeneration precedes it. Compared to muscle and ligament damage, the present findings suggest that facet joint damage plays only a minor role in the multifactorial pathogenesis of ASDeg.

### Key takeways

1. *Clinical literature suggest that iatrogenic facet joint damage correlates with increased rates of ASDeg.*
2. *Although biomechanical and *in silico* studies emphasize the importance of the facet joints for segmental stability, superior facet joint violations are unlikely to lead to adjacent segmental instability as hypertrophic or arthritic facet joints seem to be associated with reduced motion.*
3. *Increased rates of ASDis are more likely due to painful soft tissue impingement or irritating implant contact.*
4. *Iatrogenic facet joint damage cannot explain the high rate of ASDeg seen in clinical practice, especially at the caudal levels. It is a contributing factor but likely not the trigger of the degenerative cascade.*

## Fusion angle

**Fig. 7:**
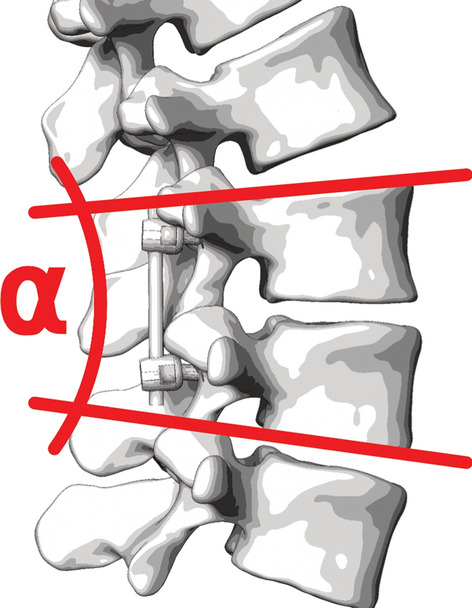
Fusion angle or segmental lordosis of index level

This section elaborates on the potential effect of the fusion angle on the biomechanics of the spine up to the development of ASDeg (Fig. 7). Particularly, this section investigates the relationship between ASDeg and segmental lordosis changes in the index and adjacent segments.

### Iatrogenic alteration

Interbody fusions (e.g., PLIF, TLIF, ALIF) are amongst the most frequently used, because of the higher rates of fusion in comparison to non-interbody spinal fusion techniques (e.g., PLF) [129]. In the process, the IVD is partially or fully resected and a spacer implant (cage) and/or a graft are placed in the emerging disc space [34]. The implant is meant to maintain or restore the vertical distance and the angle between the adjacent vertebral bodies. Altering this angle also referred to as fusion angle [130], or segmental lordosis angle (SLA), allows the surgeon to correct and adapt the lumbar lordosis with potential consequences for the whole spinal alignment. The intervertebral cage serves as a hypomochlion which allows the surgeon to set the lordotic angle by dorsal compression. The quality of intervertebral angle restoration depends on the implant size which varies with the surgical technique. Anterior approaches (e.g., ALIF) and lateral approaches (e.g., XLIF) allow a controlled placement of accordingly sized cage and graft. Posterior (e.g., PLIF, TLIF) and endoscopic approaches, on the other hand, provide only narrow access to the intervertebral space, limiting the implant size and the surgeon’s scope of action for alignment corrections [34].

### Pathomechanism

The fusion angle influences the endplate inclination of the cranial vertebra, the lumbar lordosis (LL), and such, the spinopelvic alignment. Earlier literature suggests that fusion can lead to a significant loss of lordosis, often described as “flat back syndrome” [131]. The malalignment of the fused segment could lead to adverse loading and non-uniform stress transfer in the adjacent levels. Biomechanical cadaver experiments based on radiologic patient data suggest that postoperative lordosis loss may lead to non-physiological loading of the adjacent motion segment [132]. Barrey and Darnis hypothesized that a hypolordotic arthrodesis could induce hyperextension of the cranial non-instrumented level [133]. Hyperextension results in higher compressive loading on the posterior disc and facet joints, while the anterior disc is subject to increased tensional stresses. Compressive overloading bears the risk of nerve root impingement and damage to the posterior part of the disc and facet joints. Extreme extension of the anterior disc may cause microdamage and tears in the anterior disc. Although risks for intraoperative and postoperative complications remain, the advances in surgical techniques and implant design (rod-bending, poly-axial screws, expandable cages) in combination with preoperative planning modalities help to prevent hypolordotic fusion constructs.

Risks associated with hyperlordotic fusion, on the other hand, have received little attention yet. Fusing the pathologic segment in strong lordosis may lead to a good spinal alignment in the standing posture. However, this neglects other postures encountered in everyday life. For instance, slumped sitting significantly decreases lumbar lordosis and pushes the entire spine towards a C-shaped configuration [134]. As hyperlordotic fusion prevents the straightening of the lower back, it may force the adjacent segment into hyperflexion. As European white-collar workers frequently report spending up to two-thirds of their daily time seated [135], permanent non-uniform stress distribution above the instrumented levels becomes a risk factor to be considered. Permanent hyperflexion leads to compression of the anterior disc, while its posterior part as well as the ligaments and facets joints are stretched inducing tensional stresses. Repeated overstress makes the annulus fibrosis prone to microdamage and tears, with the potential of subsequent bulging of the nucleus pulposus (disc herniation).

### State of the literature

The following literature investigated the postoperative segmental lordosis (SL) and/or pre- and postoperative differences in segmental lordosis (∆SL) in patients with and without ASDeg (Table 8, Table 9, and Appendix Table 19):

- Okuda et al. analyzed 42 ASDis patients and 54 controls with a mean age of 67 years who received single-segment PLIF at L4/5 for degenerative lumbar spondylolisthesis with at least 5 years of follow-up (8.7 years on average) [136]. Postoperative radiographs were evaluated one year after surgery. The postoperative SL did not differ significantly between the groups. However, the non-ASDis patients exhibited an increase in segmental lordosis (∆SL) of 1.1°, whereas the ASDis group had a reduction in (∆SL) by -2.4° (*p* = 0.002).
- A retrospective study focused on L4-5 posterior and transforaminal lumbar interbody fusion [137]. It compared non-ASDeg (70 patients, average age 56.7, follow-up 46.7 months) and ASDeg (19 patients, average age 52.1, follow-up 52.5 months) groups. Immediately postoperative, the non-ASD group exhibited a significantly higher SL of 10.9° ± 4.0° than the ASDeg group (8.1° ± 4.0°, p = 0.009). The change in SL (∆SL) was comparable with 13.4° ± 5.7 in the non-ASDeg and 11.3° ± 5.9° in the ASDeg group. Further, the study reported no significant difference in SL at the final follow-up.
- The outcomes of L4-5 or L5-S1 ALIF/TLIF were analyzed in a retrospective study [65]. The non-ASDeg group included 92 patients, averaging 48.7 years, with a follow-up of 58.1 months, while the ASDeg group comprised 9 (8.7%) patients averaging 46.6 years with a follow-up of 68.5 months. The non-ASDeg group had a lower postoperative SL of 16.8° ± 8.5°compared to the postoperative SL of 19.8 ± 4.9 in the ASDeg group, but the difference was not statistically significant (p = 0.093). The change in SL (∆SL) was not reported and it was noted that radiographic follow-up was available for only 87.4% of the cases.
- A retrospective study analyzing L4-5 fusion outcomes compared 18 non-ASDeg patients (mean age 56.2 years) and 30 ASDeg patients (mean age 51.6 years) over an average follow-up of 44.6 months. The postoperative SL was 15.3° ± 4.6° in the non-ASD group and 17.2° ± 6.2° in the ASDeg group. Although the change in SL (∆SL 1.0° ± 5.7°) was smaller for non-ASD than for ASDeg patients (∆SL 2.8° ± 4.9°), the differences were statistically insignificant. The study noted a significant age disparity between the groups and a high ASDeg rate due to a large amount of preexisting adjacent segment degeneration.
- Hikata et al. conducted a retrospective study on L4-5 PLIF for L4 degenerative spondylolisthesis [90]. Analyzing non-ASDeg (23 patients, average age 66.4, follow-up 53.5 months) and ASDeg/ASDis groups (31 ASDeg and 7 ASDis patients, average age 58.7, follow-up 64.6/54.8 months), it was found that the non-ASDeg group had an immediate postoperative SL of 1.1° ± 5.6°. Although the ASDis group had a postoperative SL of 6.3° ± 4.3°, the difference was not significant. However, the study highlighted a significant difference in SL at the final follow-up (*p* = 0.005).
- A prospective study on 1- or 2-level TLIF involved non-ASDeg (54 patients, average age 47.1, follow-up 60.12 months) and ASDeg groups (31 ASDeg and 15 ASDis patients, average age 54.2, follow-up 60 months or surgery) [138]. The non-ASDeg group showed an immediate postoperative SL of 13.0° ± 6.9° and the ASDis group had a postoperative SL of 13.7° ± 6.4° with no significant difference noted (*p* = 0.647). The study reported an increase in mean SL from pre- to post-surgery, indicating a correction of lordosis but no statistical analysis was provided.
- A retrospective study on single- or multi-level fusion compared 76 non-ASDeg patients (average age 67.5) and 31 ASDeg patients (average age 65.3) with an average follow-up of 27.8 months [139]. Right after surgery, the non-ASDeg group's SL was 19.0° ± 8.8° and the ASDeg group had a SL of 20.9° ± 9.0°. The ∆SL was 2.1° ± 9.3° and ∆SL of 4.5° ± 11.8°, respectively. Both postoperative SL and ∆SL showed no significant difference (*p* = 0.3).
- Matsumoto et al. conducted a retrospective matched-case control study on L4-5 PLIF in 20 ASDis patients (average age 68.9 years, follow-up 37.0 months) and 100 controls (average age 66.7 years, follow-up 68.6 months) [140]. The postoperative SL was 12.9° ± 6.9° in the control group, with no significant difference to the ASDis patiens (12.8 ± 6.2). The ∆SL data was not reported, and postoperative radiographs were evaluated at the end of follow-up.
- A retrospective study on multi-level PLIF/TLIF included a non-ASDis group (124 patients, average age 61.0 years, 14-month follow-up) and an ASDis group (13 patients, 9.5% incidence, average age 57.8 years, 41-month follow-up) [141]. The non-ASDis group showed a postoperative SL of 27.2° ± 9.0°, which was not significantly different from the ASDis group with a postoperative SL of 28.1° ± 7.9°. No ∆SL and inconsistency in postoperative follow-up periods was reported.
- In a prospective study on multi-level PLF/PLIF, Anandjiwala et al. compared 54 non-ASDeg patients and 14 ASDeg patients (overall age 43–78 years, follow-up 67.4 months) [119]. Three months after surgery, the non-ASDeg group had a SL of 19.76° ± 8.90° and the ASDeg group a SL of 20.47° ± 10.21°, showing no significant difference. The study noted that in the ASDeg group, LL increased between pre- and postoperative measurements indicating a corrective intervention.

**Table 8:** Postoperative segmental lordosis (SL) and change in segmental lordosis (∆SL) in ASDeg compared to control or non-ASDeg group (summary of Appendix Table 19)

	Postoperative SL in ASDeg group compared to control/non-ASDeg group	∆SL in ASDeg group compared to control/non-ASDeg group
Smaller (significant)	1 [137]	–
Smaller (not significant)	–	2 [71, 137]
No change	2 [119, 138]	–
Higher (not significant)	3 [65, 71, 139]	1 [139]
Higher (significant)	–	–

**Table 9:** Postoperative segmental lordosis (SL) and change in segmental lordosis (∆SL) in ASDis compared to control or non-ASDis group (summary of Appendix Table 19)

	Postoperative SL in ASDis group compared to control/non-ASDis group	∆SL in ASDis group compared to control/non-ASDis group
Smaller (significant)	–	1[136]
Smaller (not significant)	1 [136]	–
No change	2 [140, 141]	–
Higher (not significant)	1 [90]	–
Higher (significant)	–	–

In summary, the review of 10 studies examining postoperative segmental lordosis (SL) and change in segmental lordosis (ΔSL) in patients with ASDeg and ASDis compared to controls revealed heterogeneous outcomes. While 1 study reported significantly smaller postoperative SL in ASDeg groups and 1 study showed significantly smaller ΔSL in ASDis groups, the remaining studies demonstrated either no significant difference or inconsistent trends, indicating that the relationship between these radiographic parameters and the development of adjacent segment pathology remains inconclusive.

### Discussion

Although correcting the fusion angle may affect the overall lumbar lordosis, distinguishing between local and global changes in spinal alignment can be challenging and misleading. Consequently, a substantial body of literature comparing spinopelvic parameters, such as LL, SS, PT, and PI, or specific ratios thereof, in patients with ASDeg and control groups [142–150], was excluded. Instead the focus was solely on literature reporting the fusion angle (segmental lordosis).

Despite frequently reported, the sole measurement of postoperative SL lacks information about lordosis changes in the fused levels. Such insight can only be retrieved by calculating the delta between post- and preoperative SL (∆SL). From the 4 studies reporting the ∆SL, 2 found a smaller ∆SL (decrease in SL) and 1 study a higher ∆SL (increase in SL) in the ASDeg groups. However, only Okuda et al. reported statistically significant smaller ∆SL (decrease in SL) in the ASDis group [136]. Being the only study investigating ASDis patients, its findings corroborate the hypothesis that hypolordotic fusion or a loss of SL within 1 year after surgery promotes the development of ASDis. The remaining studies only partly support this hypothesis. Possible explanations are the inclusion of asymptomatic ASDeg patients and the variations in timing of postoperative radiographic evaluation. Evaluating the postoperative radiograph immediately after surgery ignores potential adaptations that occur before the completion of bony fusion. Furthermore, manual radiographic lordosis measurements are subject to inter- and intra-rater variability and show a minimal detectable change of more than 3 degrees [151], which questions the suitability to detect such small changes in SL. Here, consistent reporting using computer-guided methods may help to standardize the measurement of spinal parameters in the future [152]. Moreover, most analyses are based on static measurements, which fail to account for potential influences during active motion.

A relatively new hypothesis highlights hyperflexion of segments above normal or hyperlordotic fusions during flexion-heavy activities (e.g., sitting). Since the preoperative alignment is determined during erect standing and the fusion is executed in a supine position, the fusion construct usually neglects the possibility of sitting-induced hyperflexion of the cranial adjacent segment. On these grounds, the patient’s activity profile should be considered regarding the fusion angle and postoperative care instructions. Apart from a musculoskeletal model analyzing hyperlordotic fusion [130], biomechanical and clinical studies investigating this hypothesis are still scarce but because of the increasing frequency of hyperlordotic fusions [153, 154], this hypothesis should receive more attention in future research.

While the relationship between anatomic segments and their influence on sagittal balance is evident in healthy populations [155], the long-term impact of spinal fusion on adjacent spinal regions remains less established. The question of how muscles and ligaments can adapt and maintain sudden alignment changes remains unanswered, as the interplay between spinal balance and the musculoligamentous system is still poorly understood [156]. However, there appears to be a link between postoperative sagittal imbalance and increased long-term ASDis risk [157], which emphasizes the need to investigate the relationship between local and global spinal alignment.

Overall, determining the right fusion angle(s) and the “optimal” lordosis for the patient remains a primary challenge of pre-surgical planning. Patient-specific musculoskeletal [158] and finite element models [159] provided promising insights, and they have the potential to support surgeons in finding alignments that minimize the risk of ASDeg development. Nevertheless, based on the reviewed literature and the discussed shortcomings, the evidence for a strong impact of the fusion angle on the development of ASDeg is insufficient.

### Key takeaways

1. *Although a plethora of studies investigated the relationship of spinopelvic balance and ASDeg, the impact of the fusion angle is not well documented, as the changes in segmental lordosis were barely reported.*
2. *Those studies examining changes in segmental lordosis in patients with ASDeg have yielded inconsistent results, thereby rendering the relationship between fusion angle and ASDeg development inconclusive and demanding a consistent reporting of segmental lordosis changes in future studies.*
3. *While one study suggests hypolordotic fusion may increase ASDeg risk, overall evidence for a strong impact of a hypolordotic fusion angle on ASDeg development is considered very low. However, increasing sedentary behavior and trends towards aggressive lordosis restoration call for a thorough investigation of this hypothesis.*
4. *Preoperative planning may benefit from the integration of computational and statistical models, that facilitate the selection of an adequate fusion angle and restoration of spinal balance.*

## Immobilization

This section elaborates on the potential effect of spinal fusion on the biomechanics of adjacent segments, particularly focusing on changes in motion patterns and load distribution. It explores if the immobilization of one or more spinal segments through fusion can lead to compensatory mechanisms in the remaining mobile segments and their role in the development of ASDeg. The unique anatomy of the spine enables humans to perform complex motion patterns. Each spinal segment acts as joints with three degrees of freedom allowing flexion–extension, lateral bending, axial rotation or any combination thereof [103]. At the same time, the discs, facets, and ligaments restrict motions to protect the spinal cord from excessive strains [24]. The resulting in vitro and in vivo kinematics of the lumbar spine have been described previously [75, 160]. In addition to complex motions, daily activity subjects the spine to static (body weight) and dynamic (walking, carrying, driving), mostly vertical, compressive loading. In conjunction with the musculoligamentous system, the IVDs are believed to serve as cushions that dampen these loads and motions [161–163].

### Iatrogenic alteration

**Fig. 8:**
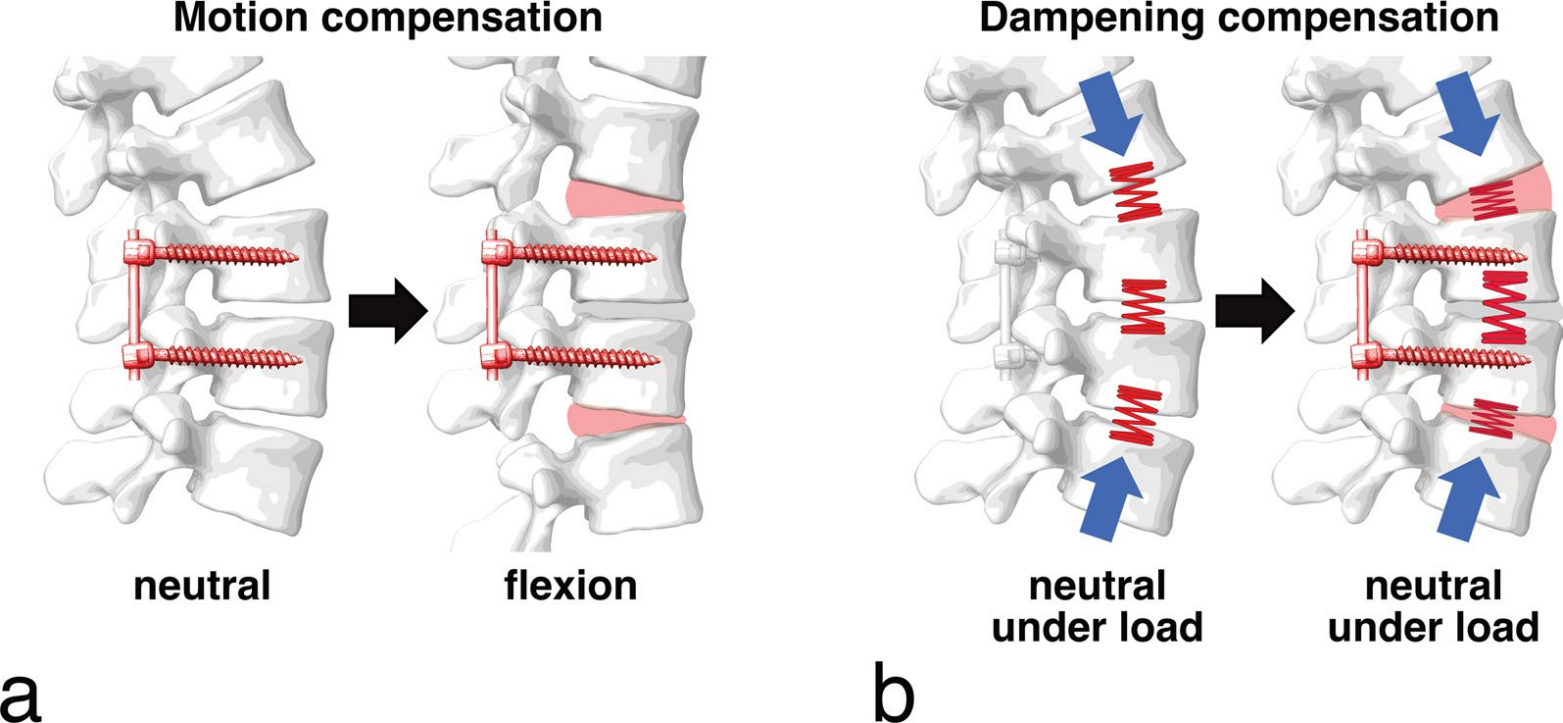
Immobilization leading to **a** motion compensation or **b** dampening compensation in adjacent segments

The goal of every fusion surgery is the immobilization (Fig. 8a) of the pathological segment to relieve the patient from symptoms and further avoid motion-induced pain and neural compression. To further restrict motion and facilitate bony fusion, the vertebral bodies of the respective segment are typically instrumented with pedicle screws and rods. On each side, the superior and inferior screw heads are connected with rods, creating a rigid construct. Following the successful arthrodesis, the fused segments become immobile, thereby restricting the mobility of the spine and potentially altering the mechanics and kinematics in the remaining mobile segments.

### Pathomechanism

#### Motion compensation

A widespread hypothesis in spine research suggests that compensatory motion in the segments adjacent to fusion plays a key role in the development of ASDeg [5, 11, 164] (Fig. 8a). Pedicle screw-rod constructs and subsequent arthrodesis restrict the motion of the index spinal segment. To reach the presurgical range of motion, the uninstrumented segments must compensate for the reduced mobility. This effect is believed to be most pronounced in the proximal adjacent segments. Therefore, the compensating segments experience larger stresses and strains in the load-bearing structures. Strains exceeding the physiological limits may increase the likelihood of irreversible damage and subsequent degeneration, especially in the IVDs and facet joints. For example, repeated or permanent hyperflexion compresses the anterior annulus and leads to higher tensional stresses in the posterior annulus, possibly causing microdamage and tears and increasing the risk of herniations. Also, the ligaments and the capsules of the facet joints would be subject to higher tensional stresses, potentially causing damage and painful inflammations. Inverse effects, such as increased anterior tension and posterior compression, are to be expected for the adjacent segment when compensating for extensional movements.

#### Dampening compensation

As the spine is deprived of one or more segments to physiologically buffer vertical loading and shocks (Fig. 8b), the axial peak loading on the remaining segments potentially increases. Given that this compressive loading regularly exceeds the physiological range, microscopic tears and lesions are to be expected. Over time, this micro-damage may accumulate and result in irreversible damage to the load-bearing soft tissues such as endplates, discs, and facet joints.

Multi-level fusions are expected to amplify both effects as fewer segments are available to absorb vertical loading or to perform the movement. Moreover, long fusion constructs alter the instantaneous center of rotation [165], and thus, potentially change the lever arms of forces acting on the adjacent segments. Altered lever arms may increase the moment and resulting stresses on the functional tissues, which again, can induce irreversible damage when exceeding the physiologically acceptable range.

### State of the literature

#### Motion compensation

Malakoutian et al. questioned whether the compensatory motion often reported in biomechanical experiments was also observed in clinical and in vivo studies [10]. Based on their findings, the review presents an updated analysis on this subject, detailing the changes in total lumbar, upper segment, and lower segment angular ROM as well as adjacent segment translational ROM. The review only includes publications reporting absolute values. Malakoutian et al. already provided a clear explanation as to why reporting relative ROM can be misleading [10].

Of the 24 studies identified, six investigated total lumbar angular ROM. All studies reported a postoperative reduction in total lumbar angular ROM, with half demonstrating statistical significance.

With regards to the delta in the upper adjacent angular ROM, eight studies reported no change or only a statistically insignificant decrease. Conversely, 15 studies identified an increase, of which 12 showed statistical significance. For the caudal adjacent segment, 10 studies found no change or a small decrease, while only one study reported increased angular ROM. The outcomes are summarized in Table 10. A detailed analysis of the literature can be found in Appendix Table 20.

**Table 10:** Change between pre- and postoperative angular ROM in the lumbar spine (T12-L1 to L5-S1), the upper adjacent and the lower adjacent segment. The studies of Kim et al. [166], Lee et al. [62], and Liu et al. [81] are listed multiple times as they reported different results depending on the fusion technique (summary of Appendix Table 20)

	Total lumbar angular ROM	Upper adjacent angular ROM	Lower adjacent angular ROM
Decrease (significant)	3 [167–169]	–	–
Decrease (not significant)	3 [170–172]	5 [52, 62, 167, 171, 173]	1 [171]
No change	–	3 [174–176]	7 [52, 167, 174–178]
Increase (not significant)	–	3 [62, 179, 180]	2 [173, 166]
Increase (significant)	–	12 [81, 168, 169, 177, 178, 166–186]	1 [184]

Three additional studies compared fusion patients with an asymptomatic cohort or decompression-only patients. The first study found no change in total lumbar ROM but a slightly increased cranial adjacent segment ROM compared to an asymptomatic control group [136]. The second study found a larger overall lumbar ROM in the control group than in patients undergoing ALIF [187]. The fusion patients also exhibited reduced ROM in the adjacent cranial and caudal segments. Seitsalo et al. compared posterior, posterolateral and anterior fusion patients with conservatively treated patients [188]. In terms of cranial adjacent segment ROM they noticed no differences between the pre- and postoperative state.

Furthermore, seven studies investigated the delta in translational ROM in the adjacent segments which describes the change in displacement parallel to the endplates of the fused vertebra (Table 11, Appendix Table 21). Of four studies, four found an increase (one significant) in upper adjacent segment olisthesis after fusion surgery. Increased caudal olisthesis was registered in three studies but without statistical significance while two studies observed no change. Although incidences ASDis were partially reported, none of the studies related the pre- to postoperative changes in ROM with the occurrence of ASDis. In conclusion, the literature suggests a reduction in total lumbar angular ROM, while upper adjacent angular ROM tends to increase, and the lower adjacent angular ROM remains largely unchanged.

**Table 11:** Change between pre- and postoperative translational ROM. The study of Jeong et al. [52] are listed multiple times as they reported different results depending on the surgical approach (summary of Appendix Table 21)

	Upper adjacent translational ROM	Lower adjacent translational ROM
Decrease (significant)	–	–
Decrease (not significant)	–	–
No change	–	2 [52, 189]
Increase (not significant)	3 [52, 180, 181]	3 [52, 180, 183]
Increase (significant)	1 [81]	–

#### Dampening compensation

To investigate whether extended fusion constructs potentially exacerbate adverse mechanical changes, a literature review was conducted, comparing the incidence of adjacent segment degeneration (ASDeg) and adjacent segment disease (ASDis) in long versus short fusion constructs (Table 12, Appendix Table 22). A total of 17 studies were identified, comprising two prospective and 15 retrospective studies, which compared single-level fusions with two- or multi-level fusions. The incidence of ASDeg was found to be higher in longer fusion constructs, as reported by seven studies. However, only two of these studies reported statistically significant differences between single-level and multi-level fusions. Of the 13 studies that reported ASDis incidences, seven found significantly higher incidences in the long fusion groups. In four studies, the differences in ASDis incidences were not statistically significant, yet they exhibited a tendency towards higher complication rates in the multilevel fusion patients. The remaining two studies showed no differences or a slightly higher incidence in the short fusion group.

**Table 12:** ASDeg and ASDis incidence in short and long fusion constructs (summary of Appendix Table 22)

	ASDeg incidence	ASDis incidence
Significantly higher in short fusion	–	–
Higher in short fusion (not significant)	–	1 [84]
No difference between short and long fusion	–	1 [117]
Higher in long fusion (not significant)	5 [139, 190–193]	4 [59, 138, 190, 191]
Significantly higher in long fusion	2 [194, 195]	7 [88, 124, 194, 196–199]

### Discussion

The reviewed literature revealed that the total lumbar ROM typically decreases following fusion surgery. There is a notable increase in upper adjacent ROM, although this is not a consistent outcome across all patients. In contrast, lower adjacent ROM remains largely unchanged. For translational ROM, only insignificant increases or no changes were reported for both upper and lower adjacent segments. The incidence of ASDeg is higher in long fusion constructs, although not always to a statistically significant degree. Generally, ASDis incidences are significantly higher in long fusion constructs but it should be noted that the reviewed literature often did not differentiate between upper and lower ASDeg and ASDis occurrences.

#### Motion compensation

The significant increases in upper adjacent ROM suggest that a compensatory mechanism appears at least in a subgroup of patients following spinal fusion. One potential explanation for the observed variability in the results is that the 12 studies measured significant ROM increases in patients undergoing PLIF with or without midline decompression or multilevel fusions. The remaining eight studies reporting a decrease or no change in the cranial adjacent segment investigated ALIF (with or without instrumentation), TLIF or posterolateral instrumented fusion. These differences suggest an impact of the surgical approach and the fusion technique on the adjacent segment ROM. The literature generally reports higher ASDeg and ASDis incidences in upper adjacent segments [124, 190], which coincides with the here reported increase in upper adjacent ROM and indirectly supports the motion compensation hypothesis. Upon fusing additional segments, the adjacent one is required to compensate to a greater extent, which may potentially elevate the probability of loading conditions exceeding the physiological limit. This consideration presupposes that a segment is already close to its limit of physiological motion. Then, even small changes in absolute range of motion (ROM) could be sufficient to introduce irreparable damage, linking fusion-induced hypermobility and the development of pathological damage to the adjacent segment. However, it should be noted that patients with either short or long fusion constructs may exhibit considerable variation in terms of their degenerative status and predisposition to further degeneration.

In contrast, the caudal adjacent segment ROM exhibited no significant changes or rather a slight decrease in postsurgical ROM. Adjacent segment translation was less reported and mostly showed insignificant increases in both cranial and caudal adjacent segments, thus, neither contradicting nor strongly supporting the motion compensation hypothesis. As suggested by Malakoutian, the overall decrease in total ROM oppose the notion that patients return to preoperative motion patterns and that adjacent segments fully compensate for the immobilized segment [10]. Furthermore, in vivo studies analyzing intervertebral flexion/extension and anterior/posterior (AP) translation on static end-range flexion and extension lateral radiographs have limitations as they neglect to assess mid-range motion, which constitutes most activities of daily living. To this date, the authors are only familiar with one study that investigated the complex postfusion 3D kinematics in the lumbar spine [165]. The study found that changes in adjacent segment motion varied across patients, but all patients maintained or increased the amount of adjacent segment slip or intervertebral flexion/extension six months after fusion. Nevertheless, the existing literature did not address the question of whether increased adjacent motion correlates with increased rates of pathological degeneration (ASDis). The increased range of motion in adjacent segments (hypermobility) is frequently utilized as a diagnostic criterion for ASDeg. Despite the estimation that between 1/4 to 1/3 of ASDeg cases progress to ASDis [200], the causality between radiographic degeneration and disease development remains inconclusive, not only in the context of ASDeg [201]. Further, the literature focused mainly on the primary adjacent levels potentially neglecting compensatory mechanisms in suprajacent segments. These limitations relativize the significance of the hypothesis in the clinical context and suggest that motion compensation seems to be primarily a factor in long fusion constructs.

#### Dampening compensation

Due to the complex structure of the spine, the impact of fusion on its dampening properties is challenging to quantify. Although studies have reported reduced disc heights in the adjacent segment [202, 203], these effects are difficult to separate from the natural progression of degeneration and do not necessarily correlate with clinical outcomes [82]. It appears logical that the immobilization of multiple segments reduces the overall dampening capacity of the spine. The significantly higher ASDis rate in long fusions may indicate that the adjacent segments compensate for this reduced capacity, thus experiencing larger stresses and faster deterioration. Proximal junctional kyphosis (PJK) and proximal junctional failure (PJF) can be seen as extreme examples of ASDeg and ASDis that demonstrate early (< 18 months) mechanical alteration and failure in the adjacent segment [204]. Although incidences of 20% to 40% are reported [204], they almost exclusively occur in adult spinal deformity patients with very long fusion constructs.

On the other hand, the correlation between higher ASDeg incidences in patients with longer fusion constructs could be associated with the dampening and shock attenuation properties of the spine. It is believed that the spinal curvature enhances the strength, flexibility, and shock-absorbing capacity of the vertebral column. Quantitative investigation of the shock-absorbing capacity of the human musculoskeletal system showed that low back pain and age correlate with a reduced shock attenuation capacity of the musculoskeletal system [163, 205]. Using an upright positional MRI scanner Meakin et al. revealed individual variations in spinal curvature response to load: spines with less curvature tended to straighten, while those with more curvature increased in curvature [206]. Another study found a significant correlation between lumbar lordosis and the spine’s shock attenuation during running, supporting the hypothesis that spinal curvature is crucial in reducing impact shocks during high-impact activities [207]. These findings also challenge the view that intervertebral discs are the primary ‘shock absorbers’ of the lumbar spine [161–163]. Although not commonly considered in the context of ASD, rigid fusion could significantly compromise the dampening function of the spine by removing one or multiple IVD “cushions” and thus increase the dynamic loading of the remaining motion segments. However, how fusion may affect the overall damping and the vertical load transfer was only partially investigated on in vitro or computational models. Axial compressive loading (up to 750 N) of six human cadaveric spine segments revealed that adjacent to a L5/S1 fusion the IDP in the L4/L5 level increased by 15% on average [208]. A poroelastic finite element model demonstrated that, during daily activities, segments adjacent to fusion experience higher strains and stresses leading to decreased disc heights and increased fluid loss [209]. The authors suggest that disc height reduction and outgoing fluid flow further debilitates the disc’s dampening and stress absorption qualities. Overall, the dampening compensation in adjacent segments might only be problematic in long fusion constructs. In these cases, the effects of motion and dampening compensation might overlap and amplify each other, potentially leading to accelerated degeneration and increased risk of ASDeg.

### Key takeaways

1. *Although there is a general decrease in total lumbar ROM, the majority of literature reported an increase in angular ROM in the cranial adjacent segment which supports the motion compensation hypothesis that excessive motion may be causative for and not the consequence of ASDeg.*
2. *In contrast, the caudal adjacent segment generally showed no significant change or a slight decrease in ROM and mostly insignificant increases in both cranial and caudal adjacent segment translation.*
3. *The incidence of ASDeg and ASDis is higher in patients who have undergone multilevel fusion procedures, which lends support to the hypothesis that long fusion constructs may compromise the dampening function of the spine, leading to overcompensation and pathological degeneration in the cranial adjacent segment.*

## Summary

The literature search, while extensive, had limitations. Focusing on only one database may have excluded relevant studies. Although Embase includes all Medline records, Embase indexes content differently. Despite the use of Emtree candidate terms, the search terms might not have captured all relevant studies due to variations in terminology. To mitigate this, reference lists of included articles were searched manually. In addition, limiting the search to English and German publications, while necessary to avoid misinterpretation, may have overlooked potentially relevant non-English/German evidence.

ASDeg is multifactorial and most probably develops from a combination of the investigated biomechanical and other non-biomechanical factors. This review has focused on summarizing the current state of knowledge about the pathomechanic pathways and classifying the underlying hypotheses according to the available evidence. Based on the gathered evidence, the authors propose to sort the hypothesis in ascending order from most (1.) to least (5.) impactful on ASDeg and ASDis development:

1. **Ligament damage**
  A substantial body of research supports the notion that iatrogenic ligament damage following spinal fusion surgery potentially promotes the occurrence of ASDis. Biomechanical and in vivo analyses have demonstrated that damage to the posterior ligaments significantly increases segmental range of motion (ROM), underscoring the critical role of these structures in adjacent segment stability. Comparisons between anterior or lateral and posterior surgical approaches suggest reduced ASDeg rates with posterior complex-sparing techniques. While preserving the posterior osteoligamentous complex also helps protect muscle attachment points, iatrogenic ligament damage itself appears to be a pivotal factor in ASDeg development. Notably, the present review revealed no studies that directly compared PLIF with TLIF, both performed with the same surgical approach (open or MIS) with respect to ASDeg incidence. Given the similarity in muscle damage and restoration quality between TLIF and PLIF, with the former reducing ligament disruption, such comparisons could help isolate the effect of sparing the posterior ligaments and clarify their protective role against ASDeg. The growing popularity of minimally invasive fusion surgery underscores the necessity for meticulous tailoring of decompression strategies, thereby emphasizing the need for more focused biomechanical studies that elucidate the ligamentous mechanisms underpinning adjacent segment stability.

2. **Muscle damage**
  The current body of literature provides substantial evidence that iatrogenic muscle damage depends on the surgical approach and has a significant impact on long-term musculoskeletal health. Muscle-sparing approaches, such as minimally invasive surgery and endoscopic techniques, are associated with superior clinical outcomes and potentially lower rates of ASDeg, though these correlations are not consistently statistically significant. The current evidence suggests that less invasive approaches may potentially mitigate the risk of developing ASDeg compared to traditional open surgeries. Nevertheless, more large-scale long-term studies comparing minimally invasive and open approaches are required to confirm these trends and to develop effective strategies for muscle-preserving surgery. The comprehensive effects of muscle damage on spinal biomechanics and subsequent ASDeg development remain complex and not fully elucidated, suggesting a multifactorial influence beyond just muscle integrity. Although muscle-sparing techniques are typically regarded as advantageous, their applicability in complex surgical contexts is constrained.

3. **Immobilization**
  While total lumbar range of motion (ROM) typically decreases post-fusion, the upper adjacent segment often exhibits increased ROM, suggesting a compensatory mechanism, particularly in patients undergoing posterior lumbar interbody fusion or multilevel fusions. In contrast, lower adjacent segment ROM and translational ROM in both upper and lower segments show minimal changes. The incidence of ASDeg and ASDis is higher in long fusion constructs, where the effects of motion and dampening compensation could overlap and amplify each other, leading to accelerated degeneration and increased risk of ASDeg. However, the causal relationship between increased adjacent segment motion and pathological degeneration remains inconclusive. Future research should employ advanced imaging and comprehensive 3D motion analysis to clarify how increased adjacent segment motion—particularly in long fusion constructs—contributes to or accelerates pathological degeneration. Further biomechanical studies are necessary to quantify how rigid fusion affects the spine’s shock-absorbing capacity, potentially amplifying biomechanical stress on remaining motion segments and accelerating degenerative processes.

4. **Fusion angle**
  The effect of the fusion angle on ASDeg and ASDis remains a subject of debate in spinal fusion surgery. While some studies suggest a correlation between hypolordotic fusion or loss of segmental lordosis and the development of ASDis, the overall evidence for a strong impact of fusion angle on ASDis development is considered very low. The inconsistent findings may be due to variations in study design, the inclusion of asymptomatic ASDeg patients, and differences in timing of postoperative radiograph evaluation and varying measurement methods. Therefore, standardized measurements and sufficiently powered studies are needed to determine whether the fusion angle has a significant effect on the development of ASDeg. A relatively new hypothesis proposes that hyperflexion of segments above normal or hyperlordotic fusions during flexion-heavy activities may contribute to ASDeg, highlighting the need to consider patient activity profiles in surgical planning and postoperative care. Despite the apparent relationship between anatomic segments and sagittal balance in healthy populations, the long-term effects of spinal fusion on adjacent spinal regions and the adaptive capabilities of the musculoligamentous system remain poorly understood, highlighting the need for further research in this area.

5. **Facet joint damage**
  To date, the relationship between iatrogenic facet joint injuries and ASDeg in spinal fusion surgery remains a topic of ongoing research. While clinical studies suggest a correlation between facet joint damage and increased rates of ASDeg, and biomechanical studies highlight the importance of facet joints in segmental stability, the low incidence of bilateral cephalad facet joint injury (1–14% of cases) challenges the notion of a strong mechanical link between facet violations and adjacent segment instability. The occurrence of ASDeg in both cranial and caudal segments further complicates this relationship, as facet joint damage is primarily reported in upper adjacent segments. Rather than being the primary initiator of the degenerative cascade, iatrogenic facet joint damage is more likely a contributing factor to ASDeg development, with increased rates potentially attributable to painful soft tissue impingement or irritating implant contact. However, further research is required to prove this hypothesis and to investigate the facet joints’ role in proprioceptive feedback mechanisms.

Based on the reviewed literature, surgeons are encouraged to select muscle- and ligament-sparing approaches—such as the paramedian interfascial approach over the open midline approach or ventral rather than dorsal exposure when possible—to preserve paravertebral muscle planes and minimize disruption of the posterior ligamentous complex. Tubular retractors and microsurgical techniques can further reduce soft tissue trauma, and percutaneous screw placement is often beneficial in preserving muscle integrity. However, other factors must be considered, including the extent of decompression required and the surgeon's experience with minimally invasive techniques, which themselves carry drawbacks such as increased radiation exposure and longer operating times. Overall, tailoring the approach to each patient's anatomy while prioritizing minimal disruption to muscles, ligaments, and normal spinal alignment can help mitigate ASDeg and improve long-term outcomes.

## Data Availability

No datasets were generated or analysed during the current study.

## Appendix

See Tables 13, 14, 15, 16, 17, 18, 19, 20, 21 and 22.

**Table 13:** Open vs minimal invasive surgery (MIS)

Author	Year	Study type	Fusion + Instrumentation	Sample size	Age (y)	Follow-up (m)	ASDeg incidence	ASDis incidence
Mimura [45]	2021	Retro	MIS-PLIF Open-PLIF	68 32	60.2 63.3	100.5	NR	*5.9%* *18.8%**
Yee [48]	2014	Retro	MIS-TLIF Open-TLIF	52 16	62	33	8% 19%	NR
Yang [50]	2015	Retro	MIS-TLIF Open-TLIF	50 50	56.1 ± 11.0 58.0 ± 13.4	24	12% 18%	NR
Yang [49]	2018	Retro	MIS-TLIF Open-TLIF	30 30	54.7 ± 11.3 57.8 ± 11.5	60	10% 13%	NR
Zhu [44]	2018	Pro	MIS-PLIF Open-PLIF	52 49	52.1 ± 7.1 53.0 ± 6.5	94.7 ± 8.7 93.8 ± 6.9	*3.8%* *14.3%**	NR
Lin [46]	2019	Retro	MIS-TLIF Open-PLIF	34 36	65.4 ± 7.6 59.9 ± 8.2	64.8 ± 0.5 69.6 ± 0.9	*11.8%* *33.3%**	NR
Radcliff [53]	2014	Retro	MIS-ALIF Open-ALIF	23 30	45.8	45.6	NR	30% 30%
Kwon [51]	2022	Retro	MIS-TLIF Open-TLIF	108 53	61.4 62.8	120	NR	13.0% 9.4%
Jia [47]	2023	Retro	MIS-TLIF Open-TLIF	57 64	57.7 56.0	60	*33.3%* *59.4%**	NR
Jeong [52]	2022	Retro	MIS-PLIF/TLIF Open-PLIF/TLIF	43 44	47.5 52.3	126	23.6% 24.7%	34.9% 31.8%
Babu [60]	2012	Retro	MIS Open	9 48	61.1 ± 1.1 60.1 ± 1.1	> 36	NR	*0%* *12.3%**
Seng [55]	2013	Retro	MIS-TLIF Open–TLIF	40 40	56.6 ± 1.6 56.8 ± 1.7	60	NR	10% 10%
Adogwa [56]	2015	Retro	MIS-TLIF Open-TLIF	40 108	56.6 ± 11.7 56.1 ± 10.7	24	NR	0% 1%
Archavlis [57]	2013	Retro	MIS Open	24 25	67 ± 8 68 ± 7	26	NR	4% 8%
Galetta [58]	2023	Retro	MIS-TLIF Open-TLIF	101 136	57.9 63.0	24	NR	*5.8%* *15.4%**
Ramanathan [59]	2023	Retro	MIS Open	135 562	62.3 60.5	> 60	NR	5.9% 11.6%
Street [54]	2016	Retro	MIS-PLIF Open-PLIF	103 103	54.6 59.6	24	NR	*5.8%* *14.6%**

*Pro* prospective, *Retro* retrospective, *ALIF* anterior lumbar interbody fusion, *PLIF* posterior lumbar interbody fusion, *TLIF* transforaminal interbody fusion, *Open* open approach, *MIS* minimally invasive surgery/Wiltse/ Multifidius muscle bundle approach, *NR* not reported

^***^*statistically significant differences (p* < *0.05)*

**Table 14:** Anterior/lateral/oblique (ALIF/LLIF/OLIF) vs posterior/transforaminal (PLIF/TLIF) lumbar interbody fusion

Author	Year	Study type	Fusion + Instrumentation	Sample size	Age (y)	Follow-up (m)	ASDegincidence	ASDisincidence
Li [66]	2022	Retro	OLIF + PPS TLIF + OPS	36 36	58.5 ± 7.3 59.9 ± 7.0	43.13 ± 3.24 44.42 ± 4.54	19.4% 27.8%	2.78% 5.56%
Min [61]	2007	Retro	ALIF + PPS PLIF + OPS	25 23	53	44.6	*44%* *82.6%**	*0%* *6.7%**
Wu [64]	2021	Retro	ALIF + PPS TLIF + OPS	30 40	54.6 ± 15.2 54.25 ± 15.34	42.1 ± 22.6 56.20 ± 29.91	53.3% 47.5%	0.68 SR 0.35 SR
Lee [62]	2017	Retro	ALIF + PPS LLIF + PPS PLIF + OPS	27 24 31	58.22 60.80 59.45	35.42 + 9.35	NR	*37.0%* *41.7%* *64.5%**
Bae [65]	2010	Retro	ALIF + PPS TLIF + PPS	75 28	46.4 48.7	68.5 58.1	13.3% 3.6%	NR
Lee N [68]	2017	Retro	ALIF (no PS) TLIF + OPS PLIF + OPS	26 21 30	53.1 59.4 56.5	23.8 22.7 18.8	7.7% 23.8% 13.3%	NR
Tsuji [63]	2016	Retro	ALIF (no PS) PLIF + OPS	38 34	54 56	> 60	*13%* *38%**	NR
Kotani [67]	2021	Retro	OLIF + PPS TLIF + OPS	92 50	72 70	31 57	NR	7.6% 10%

*Retro* retrospective, *ALIF* anterior lumbar interbody fusion, *LLIF* lateral lumbar interbody fusion, *OLIF* oblique lumbar interbody fusion, *PLIF* posterior lumbar interbody fusion, *TLIF* transforaminal interbody fusion, *PPS* percutaneous/Wiltse pedicle screw placement, *OPS* open pedicle screw placement, *SR* 5-year disease-free survival rate, *NR* not reported,* *statistically significant differences (p* < *0.05)*

**Table 15:** Differences in ASDeg and ASDis incidences in fusion patients with or without adjacent segment decompression

Author	Year	Study type	Fusion + Instrumentation	Laminectomy	Sample size	Age (y)	Follow-up (m)	ASDeg incidence	ASDis incidence
Miyagi [87]	2013	Retro	PLIF + OPS	AS deco No AS deco	25 (seg.) 15 (seg.)	58.6	53.2	*64.0%* *20.0%**	*36.0%* *6.7%**
Hikata [90]	2014	Retro	PLIF + OPS	AS laminectomy No AS laminectomy	37 17	65.4	55.0	*67.6%* *35.2%**	16.2% 5.9%
Gard [91]	2013	Retro	Instrumented fusion	AS laminectomy No AS laminectomy	137 34	59.3	10	No diff (p = 0.37)	NR
Zhong [86]	2017	Retro	circumferential fusion/PLF	AS deco No AS deco	23 131	58.4	28.6	*30.4%* *8.4%**	NR
Michael [89]	2019	Retro	AxiaLIF	Preop AS deco No preop AS deco	7 142	51.5	72	NR	*57%* *11.3%**
Sears [88]	2011	Retro	PLIF + OPS	Total Additional AS laminectomy	912 NR	50	63	NR	2.5% p.a. *2.4* × *risk increase**
Maragkos [85]	2020	Retro	PLF/PLIF	AS deco No AS deco	74 54	60	21	NR	29.7% 20.4% *OR 2.68**

*Retro* retrospective, *PLIF* posterior lumbar interbody fusion, *PLF* posterolateral fusion, *OPS* open pedicle screw placement, *AS* adjacent segment, *deco* decompression, *NR* not reported,* *statistically significant differences (p* < *0.05)*

**Table 16:** Differences in ASDeg and ASDis incidences in fusion patients with full laminectomy, partial laminectomy, or no laminectomy at the index level

Author	Year	Study type	Fusion + Instrumentation	Index level laminectomy	Sample size	Age (y)	Follow-up (m)	ASDeg incidence	ASDis incidence
Liu [81]	2013	Pro	L4-5 PLIF + OPS	Facet joint resection Semi-laminectomy Laminectomy	40 40 40	57 ± 4.2 59 ± 5.6 59 ± 5.0	68.9 ± 0.9 70.8 ± 0.7 72.0 ± 0.8	*7.5%* *10%* *43%**	NR
Ma [83]	2019	Retro	PLIF + OPS	No laminectomy Full laminectomy	22 49	60.6 ± 9.0 63.0 ± 8.6	21.1 ± 3.5 21.1 ± 2.8	*22.73%* *48.98%**	0%
Ekman [82]	2009	Pro	PLF	No laminectomy Full laminectomy	16 47	39	151.2	*12.5%* *46.8%**	NR
Heo [84]	2015	Retro	PLF/PLIF	Subtotal laminectomy Full laminectomy	287 91	58.9	71.8	NR	*5.2%* *19.8%**

*Pro* prospective, *Retro* retrospective, *PLIF* posterior lumbar interbody fusion, *PLF* posterolateral fusion, *OPS* open pedicle screw placement, *NR* not reported,* *statistically significant differences (p* < *0.05)*

**Table 17:** Intraoperative facet joint violations in the superior adjacent segment

Author	Year	Study type	Fusion + Instrumentation	Superior facet joint violation	Sample size	Age (y)	Follow-up (m)	ASDeg incidence	ASDis incidence
Bagheri [116]	2019	Retro	PLF	Yes	96	61.37	51 + 2.2	NR	*57%*
				No	534	62.37	52 + 2.3		*13%**
Oh [114]	2021	Retro	PLF/PLIF	Yes	56	64.8	164.4	*55%*	NR
				No	31			*42%**	
Choi [115]	2014	Retro	ALIF + PPS	Yes	24	NR	134.2	33%	NR
				No	25			44%	
Wang [117]	2017	Retro	PLIF/TLIF + OPS	Yes	48	54	30	NR	*25%*
				No	189				*1.6%**
Levin [118]	2018	Retro	LF	Yes	112	62.5	36	NR	*19.6%*
				No	128	64.1			*9.4%**

*Retro* retrospective, *PLIF* posterior lumbar interbody fusion, *PLF* posterolateral fusion, *ALIF* anterior lumbar interbody fusion, *LF* lumbar fusion, *OPS* open pedicle screw placement, *PPS* percutaneous pedicle screw placement, *NR* not reported, **statistically significant differences (p* < *0.05)*

**Table 18:** Facet joint sparing techniques

Author	Year	Study type	Fusion + instrumentation	Group	Sample size	Age (y)	Follow-up (m)	ASDegincidence	ASDisincidence
He [120]	2014	Pro	PLF + OPS	Facet sparing	91	45.5	> 108	*52%*	NR
				Facet abutting	87			*72%**	
Sakaura [121]	2020	Retro	TLIF + OPS	CBT TT	102	67.5	> 36	*3%*	1 (1.0%)
					77	66.4		*42%**	3 (3.9%)
Anandjiwala [119]	2011	Pro	PLIF/PLF + OPS	Facet sparing	20	63.6	67.4	4 (25%)	NR
				Facet abutting	48			10 (21%)	

*Pro* prospective, *Retro* retrospective, *PLIF* posterior lumbar interbody fusion, *PLF* posterolateral fusion, *TLIF* transforaminal lumbar interbody fusion, *OPS* open pedicle screw placement, *TT* traditional pedicle screw trajectory, *CBT* cortical bone trajectory, *NR* not reported

^***^*statistically significant differences (p* < *0.05)*

**Table 19:** Comparison of postoperative segmental lordosis (SL) and change in postoperative segmental lordosis (∆SL) between ASDeg/ASDis patients and control

Author	Year	Study type	Fusion TYPE	Group	Sample size (incidence)	Age (y)	Follow-up (m)	Postoperative SL (°)	∆SL (°)	Radio-graphic follow-up (m)
Okuda [136]	2021	Retro	1-level PLIF	Control (non-ASDis)	54/256	67	> 120	11.8	*1.1*	12
				ASDis	42/256 (16.4%)	67	8.7 (5–14)	10.8	* − 2.4**	12
Tian [137]	2018	Retro	L4-5 PLIF or TLIF	Non-ASDeg	70	56.7 ± 13.1	46.7 ± 14.0	*10.9* ± *4.0*	13.4 ± 5.7	0
				ASDeg	19	52.1 ± 16.7	52.5 ± 16.1	*8.1* ± *4.0**	11.3 ± 5.9	0
Bae [65]	2010	Retro	L4-5 or L5-S1 MIS-ALIF/TLIF	Non-ASDeg	92	48.7	58.1	*16.8* ± *8.5*/NR	NR	50.1 (mean)
				ASDeg/ASDis	9(8.7%)/ 2(1.9%)	46.4	68.5	*19.8* ± *4.9**/NR	NR	66.4 (mean)
Min [71]	2008	Retro	L4-5 fusion	Non-ASDeg	18	56.2 ± 9.6	44.6 (24–68)	15.3 ± 4.6	2.8 ± 4.9	44.6 (24–68)
				ASDeg incl. progression	30 (62.5%)	51.6 ± 7.7*	44.6 (24–68)	17.2 ± 6.2	1.0 ± 5.7	44.6 (24–68)
Hikata [90]	2014	Retro	L4-5 PLIF	Non-ASDeg	23	66.4	53.5	1.1 ± 5.6	NR	0
				ASDeg/ASDis	31(57.4%)/7(13.0%)	58.7	64.6/54.8	-/6.3 ± 4.3	NR	0
Kiss [138]	2022	Pro	1- or 2-level TLIF	Non-ASDeg	54	47.1 ± 11.6	60.12 ± 5.4	13.0 ± 6.9	NR	0
				ASDeg/ASDis	31(36.4%)/15(17.6%)	54.2 ± 10.4*	60/32.28 ± 24.48	13.7 ± 6.4	NR	0
Moreau [139]	2016	Retro	1- or multi-level fusion	Non-ASDeg	76	67.5 ± 9.5	27.8 ± 15.2	19.0 ± 8.8	2.1 ± 9.3	0
				ASDeg	31(29%)	65.3 ± 11.8	27.8 ± 15.2	20.9 ± 9.0	4.5 ± 11.8	0
Matsumoto [140]	2017	Retro	L4-5 PLIF	Control	100	66.7 ± 7.5	68.6 (36–120)	12.9 ± 6.9	NR	68.6 (36–120)
				ASDis	20	68.9 ± 9.9	37.0 (3–77)	12.8 ± 6.2	NR	37.0 (3–77)
Alentado [141]	2016	Retro	Multi-level PLIF/TLIF	Non-ASDis	124	61.0 ± 12.1	14.0	27.2 ± 9.0	NR	14.0
				ASDis	13 (9.5%)	57.8 ± 12.9	41.0	28.1 ± 7.9	NR	21.1
Anandjiwala [119]	2011	Pro	Multi-level PLF/PLIF	Non-ASDeg	54	63.6 (43–78)	67.4 (61–78)	19.76 ± 8.90	NR	3
				ASDeg	14 (20.6%)	63.6 (43–78)	67.4 (61–78)	20.47 ± 10.21	NR	3

*Pro* prospective, *Retro* retrospective, *PLIF* posterior lumbar interbody fusion, *PLF* posterolateral fusion, *TLIF* transforaminal lumbar interbody fusion, *MIS* minimally invasive surgery, *NR* not reported

^***^*statistically significant differences (p* < *0.05)*

**Table 20:** Total lumbar angular range of motion (ROM) and angular ROM in segments adjacent to index level

Author	Year	Study type	Fusion type	Group	Sample size	Age (y)	Follow-up (m)	Total lumbar ROM	Upper segment ROM	Lower segment ROM	ASDeg incidence	ASDis incidence
Auerbach [170]	2009	Pro	Circumferential lumbar arthrodesis	L4–L5	15	41.1 ± 7.1	24	Preop: 31.7 ± 20.9°	NR	NR	NR	NR
								postop: 27.9 ± 19.2°				
				L5–S1	30	39.9 ± 7.0	24	Preop: 36.9 ± 16.7°	NR	NR		
								postop: 30.9 ± 13.7°				
Cakir [167]	2009	Retro	ALIF with posterior instrumentation	L4-5	15	NR	NR	*Preop: 23.6* ± *16.2*	Preop: 7.1 ± 7.0	Preop: 5.2 ± 4.1	NR	NR
								*postop: 12.7* ± *7.1** *(L2-S1)*				
									postop: 4.2 ± 4.2	postop:5.1 ± 4.7		
Chou [171]	2002	Retro	Posterolateral fusion		32	> 60	56 (48–66)	Preop: 40°	Preop: 7°	Preop: 14°	6 (18.8%)	NR
								Postop: 28°(T12-S1)				
									Postop:6°	Postop: 5°		
Kamioka [172]	1990	Retro	Fusion with lumbar trapezoid plate		26	49 (12–67)	29 (7–56)	preop: 35°	(Relative only)	(Relative ony)	NR	NR
								postop: 27°				
Korovessis [181]	2009	Pro	1–3 level fusion		21	64 ± 11	54 ± 6	NR	*Increase**	NR	6 (28.6%)	3 (14%)
Berg [177]	2011	RCT	1- or 2-level fusion		61	38.5 ± 7.8	24	NR	L3-4: *∆4.3* ± *5.2**	NR	NR	NR
									L4-5: *∆6.0* ± *7.9**	NR		
									–	L5-S1: ∆-0.8 ± 5.1		
Kong [178]	2007	Retro	PLIF	L4-5	24	56 (38—78)	12	NR	Preop:* 7.2* ± *4.1*	Preop: 11.2 ± 5.8	NR	NR
									Postop:* 10.5* ± *5.2**	postop: 10.2 ± 7.6		
Ogawa [182]	2009	Pro	1–3 level posterolateral fusion with instrumentation		27	66.7	40.0 ± 1.1	NR	Preop: *5.6* ± *1.1*	NR	NR	2 (7.4%)
									Postop: *8.4* ± *1.1**			
Kim [178]	2009	Retro	ALIF (no instrumentation)	L4-5	14	43.4 (22 – 57)	24	–	Preop: 6.9 ± 3.2	Preop: 9.7 ± 6.4	0%	NR
								–	Postop: 7.7 ± 4.5	Postop: 10.3 ± 5.6		
			PLIF (with instrumentation)		14	45.0 (21 – 57)	24	–	Preop: *6.9* ± *2.8*	Preop: 9.2 ± 6.1	0%	
								–	Postop: *11.6* ± *3.1**	Postop: 12.0 ± 4.5		
Liu [183]	2012	Retro	PLIF	L5-S1	42		23.7 (12–38)	NR	Preop: *5.4**	NR	6 (14.3%)	NR
									Postop: *9.1**			
Zigler [180]	2012	Pro	Circumferential fusion	L3-4	1	40.5 ± 8.0	60	NR	Preop: 6.7 ± 4.6	9.1 ± 5.7	12 (28.6%)	NR
				L4-5	12				Postop: 8.5 ± 6.0	10.0 ± 4.2		
				L5-S1	30							
Cunningham [176]	2008	Pro	ALIF (threaded fusion cage BAK)	L4-5	32		24	NR	≈ ∆-0.3	≈ ∆0.1	NR	NR
				L5-S1				–	≈ ∆-0.1			
Guyer [175]	2009	Pro	ALIF (threaded fusion cage BAK)	L4-5	43	38.8 ± 8.69	60	–	No change	No change	NR	NR
				L5-S1				–	No change			
Cheng [184]	2022	Retro	PLIF	L4-5	9	40.44 ± 6.39	67.9 ± 4.88	–	Preop: *4.8* ± *2.2* (reference)	Preop: *5.0* ± *2.0*	55.6%	NR
								–	Postop (1 year): 6.7 ± 2.9	Postop (1 year): *7.5* ± *1.6**		
								–	Postop (final FU): *8.8* ± *2.4**	Postop (final FU): *8.6* ± *1.2* *		
Ohtonari [179]	2020	Retro	PLIF		14	67.8 ± 9.3	16.9 ± 9.61	–	Preop: *3.4* ± *4.1**	NR	NR	NR
								–	Postop: *5.6* ± *2.8**			
Lee [62]	2017	Retro	ALIF	L5-S1	77	56.1 (20—80)	21.6 (12–84)	NR	Pre: 9.67 ± 3.97	NR	NR	1 (3.8%)
									post: 9.09 ± 3.85			
			TLIF					–	Preop: 9.66 ± 3.89			0%
									postop: 8.67 ± 3.60			
			PLIF					–	Preop: 8.97 ± 3.83			0%
									postop: 10.02 ± 4.33			
Jeong [52]	2022	Retro	Open PLIF/TLIF	L4-5	44	47.45 ± 10.75	126 (120–168)		Decrease (12-120 m)	No change (12 m), decrease (60-120 m)	NR	33.3%
			MIS PLIF/TLIF		43	52.26 ± 14.19	121.9 (120–156)		Decrease (12-120 m)	No change (12 m), decrease (60-120 m)		
Ji [168]	2020	Retro	PLIF	L3-5	21	61 ± 6.50	25.18 ± 4.75	*Decrease**	*Increase**	NR	NR	NR
Kuo [174]	2018	Retro	TLIF	L4-5	23	58.0 ± 11.7	35.2 (24 – 89)	NR	Preop: 3.7 ± 4.4	3.7 ± 4.4	17 (37.0%)	NR
									Postop: 3.3 ± 3.7	3.3 ± 4.2		
Liu [81]	2013	RCT	A: fusion + facet joint resection	L4-5	40	57 ± 4.2	5.8 ± 0.9	NR	Preop: 5.2 ± 3.2, postop: 6.3 ± 2.8	NR	3 (12%)	NR
			B: fusion + semilaminectomy		40	59 ± 5.6	5.9 ± 0.7		Pre: 5.60 ± 2.9, postop: 6.70 ± 2.4		4 (17%)	
			C: fusion + full laminectomy		40	59 ± 5.0	6.0 ± 0.8		Preop*: 5.1* ± *3.0* postop: *8.6* ± *3.1**		17 (71%)	
Zhang [185]	2016	Retro	PLIF	Multilevel	50	52.3 ± 12.9	55.2 ± 6.8	NR	Preop: *8.5* ± *2.1*	NR	15 (30%)	1 (2%)
								–	Postop: *13.1* ± *3.1**	–		
Imagama [173]	2009	Retro	L4-5 PLIF + L3-4 decompression	L4-5	35	64.0 ± 8.9	46.8 ± 19.2	NR	Preop (L3-4): 8.2 ± 4.2	6.2 ± 3.2	L3-4: 18 (51.4%)	NR
									Postop (L3-4): 7.2 ± 5.6			
									Preop (L2-3): 6.0 ± 4.6	8.6 ± 3.0		
									Postop (L2-3): 7.0 ± 5.3			
Wu [186]	2017	Retro	PLIF	Multilevel	31	52.5 (38 – 77)	52.8	NR	Preop: *7.01* ± *1.83*	NR	NR	1 (3.2%)
									3 m post: *7.95* ± *2.35**			
									Final FU: *10.86* ± *2.09**			
Zhang [169]	2017	Retro	TLIF	L4-S1	29	50.10 ± 1.77	29.90 ± 0.78	Preop: *37.2* ± *10.2*	Preop: *8.04* ± *2.90*	NR	9 (31.0%)	NR
								Postop: *26.2* ± *4.8**	Postop: *11.06* ± *5.20**	–		
Okuda^#^ [136]	2021	Retro	1-level PLIF	L4-5	42			0.4	*∆2.5**	NR	NR	NR
				Control	54	67 (41 – 83)		− 0.4	*∆0.8*	–		
Luk^#^ [187]	1995	Retro	ALIF	Asymptomatic controls	30	37	3	54°	Reference	Reference	–	
				L4-5	32	37	5	31°	*Decreased**	*Decreased**	0%	0%
				L4-S1	20	38	7	22°	Decreased		0%	0%
Seitsalo^#^* [188]	1997	Retro	PLF/ALIF	L3-S1	13	13.8 (8—19)	171.6 (120–228)	NR	No significant difference to control and conservative-treated groups	NR	NR	NR
				L4-S1	84			–				
				L5-S1	48							

*Pro* prospective, *Retro* retrospective, *RCT* randomized controlled trial, *PLIF* posterior lumbar interbody fusion, *PLF* posterolateral fusion, *TLIF* transforaminal lumbar interbody fusion, *ALIF* anterior lumbar interbody fusion, *Open* open approach, *MIS* minimally invasive surgery, *NR* not reported

^***^*statistically significant differences (p* < *0.05)*

^*#*^*comparison to asymptomatic fusion patients or (conservatively treated) control group*

**Table 21:** Translational range of motion (ROM) in segments adjacent to index level

Author	Year	Study type	Fusion Type	Group	Sample size	Age (y)	Follow-up (m)	Upper segment olisthesis	Lower segment olisthesis	ASDeg incidence	ASDis incidence
Liu [183]	2012	Retro	PLIF	L5-S1	42		23.7 (12–38)	NR	preop: 5.4 ± 0.9 + 1.3 ± 0.4	NR	6 (14.3%)
								–	postop: 8.1 ± 1.3 + 5.4 ± 1.5		
Zigler [180]	2012	Pro	Circumferential fusion	L3-4	1	40.5 ± 8.0	60	preop: 1.3 ± 1.1	preop: 0.6 ± 0.5	12 (28.6%)	NR
				L4-5	12			postop: 1.6 ± 1.3	postop: 0.9 ± 0.5		
				L5-S1	30						
Axelsson [189]	2007	Case	Posterolateral fusion or ALIF	L4-5	9	NR	60	NR	no change (5: increase, 4: no increase)	NR	NR
				L4-S1							
				L5-S1							
Korovessis [181]	2009	Pro	1–3 level fusion		21	64 ± 11	54 ± 6	increase	NR	6 (28.6%)	3 (14%)
Jeong [52]	2022	Retro	Open	L4-5	87	47.45 ± 10.75	126 (120–168)	increase (12-120 m)	no change (12-120 m)	NR	33.3%
			MIS			52.26 ± 14.19	121.92 (120–156)	NR	increase (12-120 m)		
Liu [81]	2013	RCT	A: fusion + facet joint resection	L4-5	40	57 ± 4.2	5.8 ± 0.9	preop: *0.22* ± *0.17*	NR	3 (12%)	
								postop: *0.90* ± *0.32**			
			B: fusion + semilaminectomy		40	59 ± 5.6	5.9 ± 0.7	preop: *0.19* ± *0.21*		4 (17%)	
								postop: *0.75* ± *0.28**			
			C: fusion + full laminectomy		40	59 ± 5.0	6.0 ± 0.8	preop: *0.21* ± *0.26*		17 (71%)	
								post: *2.40* ± *0.45**			

*Pro* prospective, *Retro* retrospective, *RCT* randomized controlled trial, *Case* case control study, *PLIF* posterior lumbar interbody fusion, *ALIF* anterior lumbar interbody fusion, *Open* open approach, *MIS* minimally invasive surgery, *NR* not reported

^***^*statistically significant differences (p* < *0.05)*

**Table 22:** Fusion length

Author	Year	Design	Fusion type	Fusion l.ength	Sample size	Age (y)	Follow-up (m)	ASDeg incidence	ASDis incidence
Aiki [196]	2005	Retro	Posterior lumbar fusion	1	103	51 (16–75)	84 (24–204)	NR	*6 (5.8%)*
				> 1	14				*3 (21.4%)**
Cho [210]	2009	Retro	PLF	1	42	54	66	NR	3 (7.1%)
				> 1	39				6 (15.3%)
Galetta [58]	2023	Retro	Open-TLIF/ MIS-TLIF	1	59	58.4 (55.1 61.7)	48	NR	15 (25.4%)
				2	20	65.4 (61.2 70.0)			7 (35%)
Gillet [194]	2003	Retro	PLF	1	37	55 (22–81)	24–180	*12 (32%)*	*4 (11%)*
				2	26			8 (31%)	*7 (27%)**
				3–4	27			*18 (66%)**	*9 (33%)**
				5	10			1 (10%)	1 (10%)
Heo [84]	2015	Retro	PLIF (+ PLF)	1 (L4-5)	301	58.9 (21–82)	71.8 (24–131)	NR	30 (10.0%)
				2 (L4-S1)	77				3 (3.9%)
Kiss [138]	2022	Pro	1- or 2-level TLIF	1	61	ASD: 54.2 ± 10.4	ASD: 32.3 ± 24.5	NR	20 (32.8%)
				2	24	No ASD: 47.1 ± 11.6	No ASD: 60.1 ± 5.2		11 (45.8%)
Kobayashi [197]	2018	Retro	PLIF/TLIF	1	1006	65.9 ± 12.5	24	NR	*2 (0.2%)*
				> 1	357				*8 (2.2%)**
Li [195]	2015	Retro	PLF	1	44	62.3 ± 8.7	65.2 ± 6.5 (54–71)	*6 (13.6%)*	NR
				2	38			*10 (26.3%)**	
				3	20			*10 (50.0%)**	
Li [198]	2018	RCT	PLIF + OPS	1	47	53.2 ± 7.2	48	NR	*0%*
				> 1	46	51.8 ± 6.8			*4.3%**
Martini [199]	2020	Retro	ALIF	1	268	52.6 ± 0.8	42.7 ± 1.5	NR	*20 (7.5%)*
				2	136	53.6 ± 1.2	43.2 ± 2.1		*20 (14.7%)**
Moreau [139]	2016	Retro	ALIF/TLIF	1	73	67.5 ± 9.5	27.8 ± 15.2	16 (21.9%)	NR
				> 1	34	65.3 ± 11.8	27.8 ± 15.2	15 (44.1%)	
Okuda [124]	2018	Retro	PLIF + OPS	1	945	67 (16–87)	99.6 (24–252)	NR	*8.6%*
				2	55				*16.4%**
Park [190]	2009	Retro	PLIF	1 (L4-5)	28	56.4	60.5 (48 to 83)	10 (35.7%)	NR
				2 (L3-5)	23			15 (65.2%)	
				3 (L2-5)	16			15 (93.8%)	
Sears [88]	2011	Retro	PLIF + OPS	1	593	63 (14–92)	63.6 (6–192)	NR	*1.7% (1.3–2.2)*
				2	216				*3.6% (2.1–5.2)**
				3–4	117				*5.0% (3.3–6.7)**
Soh [191]	2013	Retro	PLF/PLIF + OPS	1	26	50	102	8 (31%)	NR
				2–3	29			13 (45%)	
Wang [117]	2017	Retro	PLIF/TLIF + OPS	1	178	53.2 ± 10.8 (37- 69)	ASD: 31.2 ± 2.4	NR	11 (6.2%)
				2	59		No ASD: 30 ± 3.6	NR	4 (6.8%)
Wang [193]	2021	Retro	Posterior lumbar fusion	1	62	58.5 (41–78)	40.1 (25–119)	20 (32.2%)	3 (4.8%)
				2	47			20 (42.6%)	7 (14.9%)
				3	17			4 (23.5%)	3 (17.6%)
Yang [192]	2008	Retro	PLF + OPS	1	112	NR	> 24	13 (12%)	NR
				2	62			9(15%)	
				> 3	43			7(16%)	

*Pro* prospective, *Retro* retrospective, *RCT* randomized controlled trial, *PLIF* posterior lumbar interbody fusion, *PLF* posterolateral fusion, *TLIF* transforaminal lumbar interbody fusion, *ALIF* anterior lumbar interbody fusion, *OPS* open pedicle screw placement, *MIS* minimally invasive surgery, *NR* not reported

^***^*statistically significant differences (p* < *0.05)*
